# COVID-19: Is reinfection possible?

**DOI:** 10.17179/excli2021-3383

**Published:** 2021-03-02

**Authors:** Aratã Oliveira Cortez Costa, Humberto de Carvalho Aragão Neto, Ana Paula Lopes Nunes, Ricardo Dias de Castro, Reinaldo Nóbrega de Almeida

**Affiliations:** 1 Psychopharmacology Laboratory, Institute of Drugs and Medicines Research, Federal University of Paraíba, João Pessoa, Brazil

**Keywords:** SARS-CoV-2, COVID-19, pandemic, reinfection, recurrence, recover

## Abstract

The COVID-19 pandemic has spread rapidly in many countries, overburdening health systems and causing numerous economic and social impacts. Most studies on the subject have focused on epidemiology, diagnosis, and treatment, however, there remains a scientific gap concerning the possibility of reinfection. The purpose of this bibliographic review is to gather information from studies aimed at this possibility, and to clarify what we know so far. It was found that in many situations cured patients are being released from hospitals, however, in some cases, the discharge criteria are not effective. Patients are presenting positive RT-PCR tests. There are several factors that might interfere so that patients cured of COVID-19 continue to test positive, and this would not necessarily represent a case of recurrence, as the test cannot differentiate the viral RNA from the complete virus, which alone is capable of causing the active infection. This review demonstrates that in order to rule out the possibility of COVID-19 reinfection in cured patients, more robust methods need to be adopted as criteria for both clinical discharge and post-hospital follow-up.

## Introduction

SARS-CoV-2, the virus responsible for COVID-19, has notably spread to different regions of the world. According to the World Health Organization (WHO), globally, 104,790,123 cases of the disease, and 2,285,048 deaths have been confirmed as of February 6, 2021 (WHO, 2021[39]) and this number is still increasing rapidly. However, the epidemiological characteristics of COVID-19 must also be analyzed from dynamic aspects that consider the prevalence and the rate of asymptomatic contamination (Peirlinck et al., 2020[27]). Underreporting can be seen in data published by the Center for Systems Science and Engineering at Johns Hopkins University (JHU), which reported 105,504,268 cases of the disease and 2,301,630 deaths (Dong et al., 2020[12]) on February 6, 2021. The data represent a million more cases of the disease. Declared as a pandemic by the WHO, COVID-19 has caused overloading, and in some cases, collapse in the healthcare systems of various countries; severely impacting the global economy, and compromising normally accepted behavioral liberties. The signs and symptoms of COVID-19 are similar to other virus caused respiratory infections, and less than half of patients with confirmed disease initially present fever (Guan et al., 2020[15]).

The majority of the existing studies on COVID-19 are focused on the epidemiology, diagnosis, and clinical aspects of patients with active infections, and little attention is being directed to post-recovery patient follow-up. In terms of clinical cure for COVID-19, patients are considered free of the disease 14 days after their last negative diagnostic test. The chances of reinfection in the cured population are still not well established, and this raises the question among researchers and health professionals.

In order to predict the chances of reinfection in cured individuals, a compartmentalized mathematical model for endemic COVID-19 which considers parameters such as quarantine, observation of procedures, behavioral changes, social isolation, controls, and eradication of the disease in the most exposed subpopulations was proposed. When the study was performed, the world presented approximately 900,000 confirmed cases in approximately 172 countries; 190,000 individuals had recovered, and 44,000deaths had occurred. The results of the model indicated no chances that recovered individuals present new infection. However, the model predicted that infection rates would continue to asymptotically increase for an extended period. The recovery rate, on the other hand, would continue to rise slowly and steadily, occurring over a long period of time. It is worth mentioning that the hypothesis of zero reinfection in the cured population has not been clinically confirmed. Public health data have revealed certain rare cases of reinfection, however, a rare type of coronavirus is suspected as the cause. Another important factor to consider is the limitation of the variability of the data available when the research was developed (Okhuese, 2020[26]). This study demonstrates the importance of mathematical models to assist strategic decisions in health care, the adoption of public policies that track infected/cured patients, and of measures to either restrict or relax social isolation practices. 

As infection and recovery rates continue to rise, the issue of greatest concern to healthcare professionals at the Centers for Disease Control and Prevention and the World Health Organization (WHO) is whether reinfection occurs in patients cured from COVID-19.

## COVID-19 Pathogenesis, Diagnostics and Control

In general, Coronaviruses (CoVs) are human and vertebrate pathogens. They are capable of infecting mammals, birds, insects, rodents, and various wild animal species, affecting not only the respiratory system (often triggering a dangerous acute respiratory syndrome), but also affecting the gastrointestinal, hepatic, cardiovascular, and central nervous systems (Rodriguez-Morales et al., 2020[28]). In late 2019, SARS-CoV-2 was isolated for the first time in the bronchoalveolar lavage fluid of three patients in Wuhan, Hubei province, China. Based on clinical manifestations, blood tests, and chest X-rays, the disease was diagnosed by the clinicians studying the case as a virus-induced pneumonia. Months before COVID-19 was officially reported, at least two different strains of SARS-CoV-2 were found to have occurred (Jin et al., 2020[19]). 

Since then, various studies have been performed and from genomic sequencing and analysis of its evolutionary tree, SARS-CoV-2 is a β-CoVs member. The CoVs family is a class of positive, enveloped, single-stranded RNA viruses with an extensive range of natural roots. These viruses can cause respiratory, enteric, hepatic, and neurological diseases. CoVs are genotypically and serologically divided into four subfamilies: α, β, γ, and δ. And human CoV infections are caused by α- and β-CoVs (Jin et al., 2020[19]).

COVID-19 patients themselves are currently the main infection source, with critically ill patients considered more contagious than mildly affected patients. Infected people who do not present symptoms, patients in incubation while remaining asymptomatic, those not presenting respiratory infection, and those in which the infectious virus has been proven to be eliminated are also potential sources of infection (Jin et al., 2020[19]). In addition, samples taken from patients who have recovered from COVID-19 show a continuously positive RT-PCR test (Lan et al., 2020[21]). This has never been seen in such a characteristic way in the history of human infectious diseases. In other words, infected people and patients, whether asymptomatic, during incubation, or having recovered from COVID-19 can pose challenges to prevention and control of the disease, and yield the observed high rate of contagion.

SARS-CoV-2 is transmitted person to person, predominantly by respiratory droplets, and potentially fecal-oral contact. Primary viral replication is presumed to occur in the mucosal epithelium of the upper respiratory tract (nasal cavity and pharynx), with greater multiplication in the lower respiratory tract and gastrointestinal mucosa; resulting in mild viremia. Few infections are controlled during this time and remain asymptomatic. Some patients also present non-respiratory symptoms, such as acute liver and heart damage, kidney failure, and diarrhea multiple organ involvement. This is due to the human angiotensin 2-converting enzyme (ACE2), a functional type I membrane protein receptor sequestered by SARS-CoV-2 for cellular entry, similar to SARS-CoV. It is widely expressed in the nasal mucosa, bronchi, lung, heart, esophagus, kidney, stomach, bladder, and ileum, and thus all of these human organs are all vulnerable to SARS-CoV-2 (Jin et al., 2020[19]).

For COVID-19 diagnosis in clinical practice, chest radiography is an important tool. Most cases of COVID-19 present similar characteristics for chest images, including bilateral distribution of irregular shadows and ground-glass opacity. Patients with SARS-CoV-2 infection present acute serological response and relevant detection reagents such as immuno-chromatography have been quickly developed. Viral diagnosis is an important part of our arsenal against COVID-19. After the initial outbreak, diagnostic tests based on detection of the viral sequence by RT-PCR or next-generation sequencing platforms soon became available. The Cas13-based SHERLOCK platform (specific high-sensitivity enzyme reporter unblocking) has been widely used to detect Zika virus (ZIKV) and dengue virus (DENV) in patient samples at concentrations as low as 1 copy per microliter and may prove effective for determining COVID-19 (Jin et al., 2020[19]).

Currently, there is no standardization of antiviral drugs, and over 200 vaccines are currently in development, with over 60 candidate vaccines being tested in clinical trials for the control of SARS-CoV-2. For clinical practice, symptom based treatment strategies are recommended. Many drugs, such as hydroxychloroquine or chloroquine present *in vitro* antiviral effect, in hospital results for COVID-19, due to their risks of collateral effects and lack of results repeatability, it has not been possible to confirm their benefits, either when used alone or in combination with antibiotics (Mellet and Pepper, 2021[23]).

Vaccination probably offers the best option for COVID-19 control. Epitopes, mRNA, and vaccines based on protein-RBD S structure have been widely proposed and initiated. But SARS-CoV-2 reactivation will remain a persistent problem. Considering the many patients, whether currently infected or previously exposed to the virus, SARS-CoV-2 reactivation represents a major public health concern in terms of global morbidity and possibly increased mortality. We currently have not found reliable markers to predict the risk of SARS-CoV-2 reactivation, nor do we possess validated tests to determine whether a particular drug or therapy might be associated with SARS-CoV-2 reactivation. This latter point has been determined by empirical experience (Sun et al., 2020[34]; Ye et al., 2020[41]).

Zhang et al. (2020[42]) demonstrated that cured patients may continue to be viral carriers for a certain period. It is also speculated that the virus may remain longer in certain tissues, such as in the digestive tissue as compared to the respiratory tract. Lastly, intermittent virus shedding may well occur in recovered patients.

## Results and Discussion

In terms of immune response, previous studies have found that SARS-CoV-2 promotes an overall reduction in T cells (Guo et al., 2019[16]), and that IL-6 levels and IL-2R expression increase considerably (Chen et al., 2020[11]). The decrease in the cellular immune response may be related to incomplete removal of the virus, which favors reinfection. It has been found that patients who relapse after hospital discharge are generally elderly, with impaired immune function, and the presence of comorbidities (Zhou et al., 2020[43]).

The principal underlying diseases that interfere with a good COVID-19 prognosis are diabetes and hypertension (Chen et al., 2020[11]). These factors contribute to prolonged hospitalizations, and patients in these conditions are more prone to reinfection (Zhou et al., 2020[43]).

Despite the improvement of some patients after treatment, factors such as old age, diminished immune function, structural lung disease, and pulmonary fibrosis promote incomplete blood circulation-perfusion. In these cases, the partially hidden virus is not completely removed, cells remain infected, but low levels of nucleic acid do not allow positive diagnostic testing results. Yet the principal aggravating factor is low immunity, where the virus can recover its infectious capacity and lead the patient to repeated illnesses (Zhou et al., 2020[43]).

**Figure 1:**
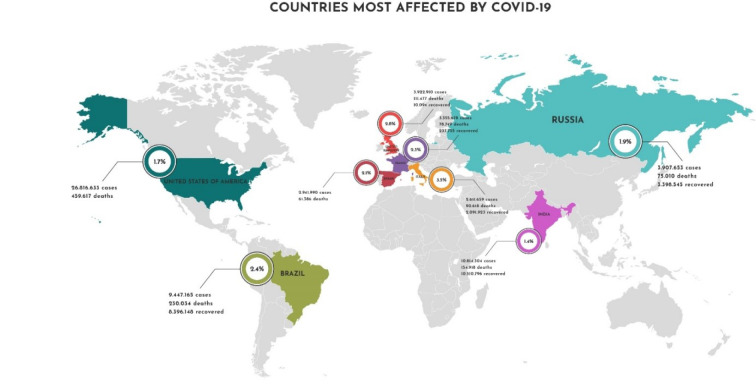
Case fatality rate of the ongoing COVID-19 pandemic. The CFR is the ratio between confirmed deaths and confirmed cases. The data used to create this figure were based on the Johns Hopkins Coronavirus Resource Center (https://coronavirus.jhu.edu), and Our World in Data (https://ourworldindata.org/coronavirus) websites. Accessed on 6^th^ February 2021

As of yet, it is difficult to estimate the SARS-CoV-2 case fatality rate (CFR), since the virus is quickly spreading. Recently, America has become the epicenter of the disease, passing Asia and Europe in numbers of infections and deaths. The CFR continues to vary widely between regions, reaching a maximum level of 18.98 % in France, in contrast to other countries like Iceland, which registered a 0.55 % CFR (Figure 1(Fig. 1)). Worldwide, the CFR is at 6.06 %, close to the profile of seasonal influenza, yet also close to the profile of other coronaviruses, such as SARS-CoV (CFR 10 %) and MERs-CoV (CFR 35 %) (WHO, 2021[39]; Biswas et al., 2020[8]). A low CFR allows the virus to continue in circulation, and with or without mutation, expanding the viral population. A high CFR implies host deaths in which entire viral populations are thus decimated, reducing both viral circulation and further potential infections (Biswas et al., 2020[8]). Figure 1(Fig. 1) shows the case fatality rate of the ongoing COVID-19 pandemic. 

Another factor that must be considered is the mutagenic capacity of the virus. Currently, the sequencing of isolated strains from different locations confirms a 99.9 % homology, with no evidence proving mutation. However, as matter of warning, an individual cured of the original SARS-CoV-2 is not effectively protected against its viral mutations or new infection (Zhou et al., 2020[43]). With SARS-CoV-2 infection, the host ends up developing humoral immunity, however, the virus' mutagenic potential may allow it to adapt and generate infections in individuals who have already recovered. The ability of SARS-CoV-2 to reinfect already cured individuals is a crucial criterion for determining whether the virus remains in circulation. Considering the possibility that mutant strains are being generated, recovered individuals should be alerted to cases of reinfection (Biswas et al., 2020[8]). Yet in normal situations, with the development of specific antibodies against SARS-CoV-2, immune individuals' chances to develop new infections from this agent are remote (Zhou et al., 2020[43]).

Bao et al. (2020[7]) conducted an *in vivo* study to investigate the possibility of reinfection by SARS-CoV-2. Challenge and re-challenge models were used with seven adult rhesus monkeys, six of whom received intrathecal administration of SARS-CoV-2 tissue-culture infectious doses (1x10^6 ^TCID_50_). The challenged animals contracted COVID-19, and after approximately 2 weeks, they were cured of the disease. Four of these monkeys were re-challenged by the same route receiving the same dose 28 days after the initial challenge. The remaining two served as the negative control of the re-challenged group. The last monkey, which was healthy, had received the initial challenge, and served as control. Clinical parameters were assessed at predetermined infection stages, including weight, temperature, chest X-ray, hematological analysis, nasal/oral/anal swabs, viral distribution, and pathological changes. Data from this study indicate that rhesus monkeys with primary SARS-CoV-2 infections cannot be reinfected by an identical strain during the initial stage of recovery. These preliminary studies are favorable and allow anticipation of positive results when conducting clinical trials for SARS-CoV-2 reinfection in humans. 

In a study conducted with seven RT-PCR positive patients for COVID-19 after symptom improvement, the patients presented two negative RT-PCR tests from throat swab samples, which is a criterion for hospital discharge. These patients remained in quarantine for 14 days, without contact with any suspected or confirmed individual, and in that period, it was observed that four patients presented positive RT-PCR from rectal swabs, two were positive in throat swabs and one was positive in both. Despite this, they were asymptomatic, and their chest CT images presented no changes when compared to the last exam before discharge (Zhang et al., 2020[42]).

Thus, reinfection by SARS-CoV-2 remains a possibility, however, the positive RT-PCR results in patients cured of COVID-19 does not necessarily reflect the recurrence of the virus, since the patients demonstrated clinical improvement before discharge, remained asymptomatic, and no imaging changes were observed. The authors indicated the need to re-evaluate hospital discharge criteria, since tests based on rectal swabs may be more reliable for guaranteeing clinical decisions; either for continued treatment or to discontinue quarantine (Zhang et al., 2020[42]).

The concern about reinfection of cured patients who leave isolation is global. An example, 116 patients recovered from COVID-19 in South Korea, and were later found to be positive. The Korean method for identifying a cured individual is based on two negative results within 24 hours. This emphasizes both the need to implement precautions before releasing the patient, and to verify whether individuals recovered from COVID-19 may or may not be infected again (Alizargar, 2020[3]).

It is worth mentioning that detection of viral RNA does not necessarily mean that the virus is present, nor that the individual has an active infection. Viral infectivity depends on the presence of the complete virus, and not only its RNA. Even if the genome is sequenced, a positive RT-PCR does not affirm viral viability (Alvarez-Moreno and Rodríguez-Morales, 2020[4]; Sah et al., 2020[30]). Certain factors need consideration: for example, a negative RT-PCR after a positive RT-PCR may occur because the viral load is below the detection threshold. Further, a positive RT-PCR after a negative RT-PCR may mean contamination. “Shedding” may be related to nucleic acid elimination deficiency in certain tissues (Alvarez-Moreno and Rodríguez-Morales, 2020[4]; Atkinson and Petersen, 2020[6]). And finally, as with the Zika virus, in some situations viral RNA can be detected long after the infection stage (Villamil-Gómez et al., 2017[38]), this, since RT-PCR is not able to differentiate between active virus and RNA (Atkinson and Petersen, 2020[6]).

To grant hospital discharge, a careful evaluation must include viral load, antibody response, detailed clinical evaluation, and follow-up. If necessary, it should be complemented with SARS-CoV-2 cell culture isolation (Alvarez-Moreno and Rodríguez-Morales, 2020[4]), but isolation depends on the viral load, and samples with less than 10^6^ copies/mL could never be isolated (Wölfel et al., 2020[40]). Any conclusions regarding cases of SARS-CoV-2 reinfection should be based on electron microscopy, genomic sequencing, and phylogenetic analysis (Alvarez-Moreno and Rodríguez-Morales, 2020[4]). 

Although positivity for SARS-CoV-2 in RT-PCR is identified in the clinic as an active infection (or of COVID-19 reinfection), studies indicate that there is a low probability of already cured individuals suffering recurrence of the disease. The research suggests that positive RT-PCR test results after cure occur largely due to the existence of viral RNA in extra-pulmonary tissues. Extra-pulmonary organs affected by SARS-CoV-2 can serve as a reservoir for the virus, contributing to viral spread after cessation of respiratory symptoms in recovered patients, and yielding false-positive test results while influencing the duration of treatment and social isolation. Patients with intermittent virus shedding may falsely be identified as reinfected. To verify clinical cure and discard the possibility of reinfection, more robust criteria need to be adopted. For Roy (2020[29]), SARS-CoV-2 reinfection is unlikely and failures in the diagnostic process, including errors in sample collection and processing techniques may explain the controversial results observed. In addition, it is necessary to assess whether the patient has actually recovered from COVID 19 or whether viral reactivation has occurred, especially in individuals undergoing glucocorticoid treatment (Alizargar, 2020[3]; Bonifácio et al., 2020[9]).

Table 1(Tab. 1) (References in Table 1: Abdallah et al., 2020[1]; AlFehaidi et al., 2020[2]; Amoozgar et al., 2020[5]; Bonifácio et. al., 2020[9]; Chan et al., 2020[10]; Duggan et al., 2021[13]; Fernandes Valente Takeda et al., 2020[14]; Hanif et al., 2020[17]; He et al., 2021[18]; Lafaie et al., 2020[20]; Lancman et al., 2020[22]; Mulder et al., 2020[24]; Nachmias et al., 2020[25]; Selvaraj et al., 2020[31]; Sharma et al., 2020[33]; Tillett et al., 2020[35]; To et al., 2020[36]; Torres et al., 2021[37]) presents case reports of SARS-CoV-2 reinfection in different countries. In general, it appears that the RT-qPCR tests are used to diagnose different moments in the course of the disease. However, studies that evaluate genetic mutations in isolated viral loads are still scarce. 

For a better view of the scientific articles published in the Pubmed database ([link:pubmed.ncbi.nlm.nih.gov*pubmed.ncbi.nlm.nih.gov]) on SARS-CoV-2 reinfection, we present their general characteristics in the Supplementary material. The references for these studies can be visualized in the Supplementary material.

Scientific evidence on this subject is scarce, and most of the published studies are non-clinical. A systematic review, which included 35 clinical and non-clinical studies, suggests that humoral immunity (if non-persistent) and errors in the diagnosis of the disease should be considered when evaluating SARS-CoV-2 reinfection (SeyedAlinaghi et al., 2020[32]). 

## Conclusion

Due to the time the virus remains in the body of the patient, positive retest results may occur. This can lead to a false diagnosis of reinfection. As well, reinfection if the virus has undergone genetic mutation is a predictable outcome. To better elucidate the problem we suggest conducting laboratory research assessing the pathogenicity of SARS-CoV-2 strains that have undergone mutations, as well as improving diagnostic records and establishing protocols for clinical cases involving possible reinfection.

## Acknowledgements

This study was supported using funds from the Coordination for the Improvement of Higher Education Personnel (CAPES), the National Council for Scientific and Technological Development (CNPq), and the Federal University of Paraíba (UFPB).

## Conflict of interest

The authors declare that the research was conducted in the absence of any commercial or financial relationships that could be construed as a potential conflict of interest.

## Supplementary Material

Supplementary material

## Figures and Tables

**Table 1:**
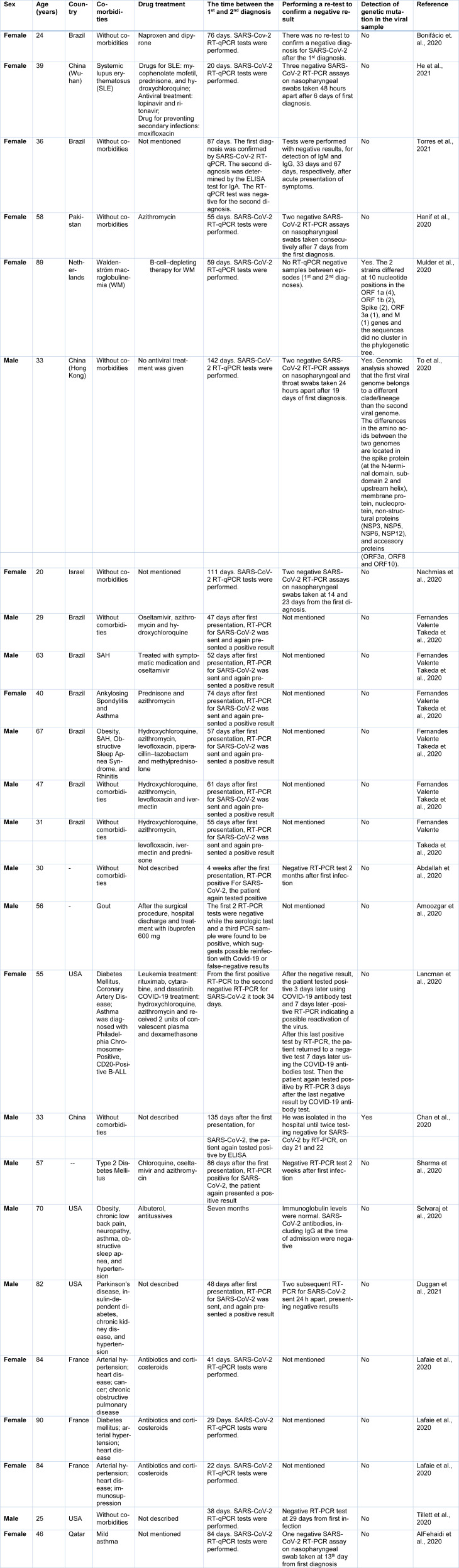
Case reports around the world of SARS-CoV-2 reinfection as described in the scientific literature
