# Supplementary material for: COVID-19: Is reinfection possible?
Source: EXCLI J. 2021 Mar 2;20:522–36. doi: 10.17179/excli2021-3383 (PMC8056061; doi:10.17179/excli2021-3383)
Supplement: Supplementary material [file EXCLI-20-522-s-001.pdf]

EXCLI Journal 2021;20:522-536 – ISSN 1611-2156
Received: January 15, 2021, accepted: February 23, 2021, published: March 02, 2021

Supplementary material to:

Review article:

COVID-19: IS REINFECTION POSSIBLE?

Aratã Oliveira Cortez Costa, Humberto de Carvalho Aragão Neto, Ana Paula Lopes Nunes, Ricardo Dias de Castro*, Reinaldo Nóbrega de Almeida

Psychopharmacology Laboratory, Institute of Drugs and Medicines Research, Federal University of Paraíba, João Pessoa, Brazil

* **Corresponding author:** Ricardo Dias de Castro, Psychopharmacology Laboratory, Institute of Drugs and Medicines Research, Health Sciences Center, Federal University of Paraíba, Campus I, 58051-970, João Pessoa, PB, Brazil, Phone +55-83-32167742. Fax: +55-83-32167175. E-mail: rcastro@ccs.ufpb.br

http://dx.doi.org/10.17179/excli2021-3383

This is an Open Access article distributed under the terms of the Creative Commons Attribution License (http://creativecommons.org/licenses/by/4.0/).

EXCLI Journal 2021;20:522-536 – ISSN 1611-2156
Received: January 15, 2021, accepted: February 23, 2021, published: March 02, 2021

Supplementary Table 1: Studies about SARS-CoV-2 reinfection published in Pubmed database* until January 30, 2021

Continent	Type of study	Number of articles	References
Europe	Original article	26	9, 13, 14, 15, 23, 47, 52, 65, 85, 86, 100, 105, 109, 118, 120, 127, 137, 143, 147, 148, 199, 204, 206, 215, 222
	Clinical case studies	10	4, 6, 39, 63, 145, 151, 152, 165, 181, 216
	Letter to the editor	14	10, 12, 42, 102, 136, 146, 155, 169, 186, 198, 207, 214, 218, 224
	Review	12	32, 49, 93, 119, 122, 176, 183, 193, 196, 212, 220, 229
	Guest editorial	6	19, 34, 58, 168, 174, 211
Asia	Original article	35	1, 5, 16, 22, 31, 38, 44, 54, 59, 61, 66, 67, 70, 74, 78, 80, 82,92, 99, 103, 107, 116, 130, 133, 134, 142, 144, 154, 166, 178, 179, 187, 190, 208, 226
	Clinical case studies	6	104, 124, 128, 203, 213, 227
	Letter to the editor	10	11, 149, 157, 158, 159, 161, 184, 195, 202, 219
	Review	11	75, 81, 84, 112, 163, 164, 173, 188, 200, 205, 223
	Guest editorial	3	35, 48, 64
Africa	Original article	7	8, 29, 45, 72, 141, 162, 177
	Clinical case studies	1	46
	Letter to the editor		
	Review	1	191
	Guest editorial		
South America	Original article	3	79, 115, 121
	Clinical case studies	7	40, 76, 90, 101, 132, 167, 170
	Letter to the editor	2	51, 197
	Review	1	28
	Guest editorial		
North America	Original article	34	2, 3, 7, 17, 20, 21, 24, 26, 30, 53, 56, 57, 68, 69, 71, 87, 89, 94,97, 98, 106, 110, 113, 117, 123, 135, 139, 150, 153, 182, 192, 209, 210, 217
	Clinical case studies	15	33, 37, 43, 55, 62, 83, 108, 129, 131, 138, 160, 175, 194, 201, 225
	Letter to the editor	5	25,73, 126, 156, 180
	Review	13	18, 27, 41, 77, 88, 91, 95, 114, 125, 172, 185, 189, 228
	Guest editorial	1	221
Central America	Original article	1	36
	Clinical case studies		
	Letter to the editor		
	Review		
	Guest editorial		
Oceania	Original article	2	60, 111
	Clinical case studies		
	Letter to the editor		
	Review	1	50

* Website: https://pubmed.ncbi.nlm.nih.gov/. Search strategy: (covid reinfection) OR (covid 19 reinfection) OR (covid-19 reinfection)

EXCLI Journal 2021;20:522-536 – ISSN 1611-2156
Received: January 15, 2021, accepted: February 23, 2021, published: March 02, 2021

REFERENCES CITED IN THE TABLE:

1. Pal R, Sachdeva N, Mukherjee S, Suri V, Zohmangaihi D, Ram S, Puri GD, Bhalla A, Soni SL, Pandey N, Bhansali A, Bhadada SK. Impaired anti-SARS-CoV-2 antibody response in non-severe COVID-19 patients with diabetes mellitus: A preliminary report. Diabetes Metab Syndr. 2020 Dec 25;15(1):193-196. doi: 10.1016/j.dsx.2020.12.035. Epub ahead of print. PMID: 33385765; PMCID: PMC7762626.
2. Gorse GJ, Donovan MM, Patel GB. Antibodies to coronaviruses are higher in older compared with younger adults and binding antibodies are more sensitive than neutralizing antibodies in identifying coronavirus-associated illnesses. J Med Virol. 2020 May;92(5):512-517. doi: 10.1002/jmv.25715. Epub 2020 Mar 3. PMID: 32073157; PMCID: PMC7166442.
3. Chaturvedi R, Naidu R, Sheth S, Chakravarthy K. Efficacy of Serology Testing in Predicting Reinfection in Patients With SARS-CoV-2. Disaster Med Public Health Prep. 2020 Jun 24:1-3. doi: 10.1017/dmp.2020.216. Epub ahead of print. PMID: 32576315; PMCID: PMC7411469.
4. Tomassini S, Kotecha D, Bird PW, Folwell A, Biju S, Tang JW. Setting the criteria for SARS-CoV-2 reinfection - six possible cases. J Infect. 2020 Aug 12:S0163-4453(20)30546-6. doi: 10.1016/j.jinf.2020.08.011. Epub ahead of print. PMID: 32800801; PMCID: PMC7422822.
5. Yahav D, Yelin D, Eckerle I, Eberhardt CS, Wang J, Cao B, Kaiser L. Definitions for coronavirus disease 2019 reinfection, relapse and PCR re-positivity. Clin Microbiol Infect. 2020 Dec 5:S1198-743X(20)30724-2. doi: 10.1016/j.cmi.2020.11.028. Epub ahead of print. PMID: 33285276; PMCID: PMC7718119.
6. Self WH, Tenforde MW, Stubblefield WB, Feldstein LR, Steingrub JS, Shapiro NI, Ginde AA, Prekker ME, Brown SM, Peltan ID, Gong MN, Aboodi MS, Khan A, Exline MC, Files DC, Gibbs KW, Lindsell CJ, Rice TW, Jones ID, Halasa N, Talbot HK, Grijalva CG, Casey JD, Hager DN, Qadir N, Henning DJ, Coughlin MM, Schiffer J, Semenova V, Li H, Thornburg NJ, Patel MM; CDC COVID-19 Response Team; IVY Network. Decline in SARS-CoV-2 Antibodies After Mild Infection Among Frontline Health Care Personnel in a Multistate Hospital Network - 12 States, April-August 2020. MMWR Morb Mortal Wkly Rep. 2020 Nov 27;69(47):1762-1766. doi: 10.15585/mmwr.mm6947a2. PMID: 33237893; PMCID: PMC7727600.
7. Dan J, Mehta S. SARS-CoV-2 immunity and reinfection. Clin Infect Dis. 2021 Jan 2:ciaa1936. doi: 10.1093/cid/ciaa1936. Epub ahead of print. PMID: 33386403; PMCID: PMC7799243.
8. Cheryl Cohen: tracking respiratory diseases, informing policy. Bull World Health Organ. 2020 Dec 1;98(12):828-829. doi: 10.2471/BLT.20.031220. PMID: 33293742; PMCID: PMC7716097.
9. Lucas TCD, Pollington TM, Davis EL, Hollingsworth TD. Responsible modelling: Unit testing for infectious disease epidemiology. Epidemics. 2020 Dec;33:100425. doi: 10.1016/j.epidem.2020.100425. Epub 2020 Nov 26. PMID: 33307443; PMCID: PMC7690327.
10. Hanrath AT, Payne BAI, Duncan CJA. Prior SARS-CoV-2 infection is associated with protection against symptomatic reinfection. J Infect. 2020 Dec 26:S0163-4453(20)30781-7. doi: 10.1016/j.jinf.2020.12.023. Epub ahead of print. PMID: 33373652; PMCID: PMC7832116.
11. Gupta V, Bhoyar RC, Jain A, Srivastava S, Upadhayay R, Imran M, Jolly B, Divakar MK, Sharma D, Sehgal P, Ranjan G, Gupta R, Scaria V, Sivasubbu S. Asymptomatic reinfection in two healthcare workers from India with genetically distinct SARS-CoV-2. Clin Infect Dis. 2020 Sep 23:ciaa1451. doi: 10.1093/cid/ciaa1451. Epub ahead of print. PMID: 32964927; PMCID: PMC7543380.
12. Liew CH, Flaherty GT. Immunity passports to travel during the COVID-19 pandemic: controversies and public health risks. J Public Health (Oxf). 2020 Aug 5:fdaa125. doi: 10.1093/pubmed/fdaa125. Epub ahead of print. PMID: 32756915; PMCID: PMC7454809.
13. McMahon A, Robb NC. Reinfection with SARS-CoV-2: Discrete SIR (Susceptible, Infected, Recovered) Modeling Using Empirical Infection Data. JMIR Public Health Surveill. 2020 Nov 16;6(4):e21168. doi: 10.2196/21168. PMID: 33052872; PMCID: PMC7674142.
14. Addie DD, Curran S, Bellini F, Crowe B, Sheehan E, Ukrainchuk L, Decaro N. Oral Mutian®X stopped faecal feline coronavirus shedding by naturally infected cats. Res Vet Sci. 2020 Jun;130:222-229. doi: 10.1016/j.rvsc.2020.02.012. Epub 2020 Feb 19. PMID: 32220667; PMCID: PMC7102653.
15. Martínez-Álvarez F, Asencio-Cortés G, Torres JF, Gutiérrez-Avilés D, Melgar-García L, Pérez-Chacón R, Rubio-Escudero C, Riquelme JC, Troncoso A. Coronavirus Optimization Algorithm: A Bioinspired Metaheuristic Based on the COVID-19 Propagation Model. Big Data. 2020 Aug;8(4):308-322. doi: 10.1089/big.2020.0051. Epub 2020 Jul 22. PMID: 32716641.

16. Xu G, Liu F, Ye M, Zhao J, Li Q, Feng C, Hu Y, Li Y, Shi H, Zhang F, Tong Y, Ma W. No Evidence of Re-infection or Person-to-Person Transmission in Cured COVID-19 Patients in Guangzhou, a Retrospective Observational Study. Front Med (Lausanne). 2020 Nov 30;7:593133. doi: 10.3389/fmed.2020.593133. PMID: 33330554; PMCID: PMC7734204.
17. Galanti M, Shaman J. Direct Observation of Repeated Infections With Endemic Coronaviruses. J Infect Dis. 2020 Jul 7:jiaa392. doi: 10.1093/infdis/jiaa392. Epub ahead of print. PMID: 32692346; PMCID: PMC7454749.
18. Babiker A, Marvil C, Waggoner JJ, Collins M, Piantadosi A. The Importance and Challenges of Identifying SARS-CoV-2 Reinfections. J Clin Microbiol. 2020 Dec 23:JCM.02769-20. doi: 10.1128/JCM.02769-20. Epub ahead of print. PMID: 33361342.
19. de Vrieze J. Reinfections, still rare, provide clues on immunity. Science. 2020 Nov 20;370(6519):895-897. doi: 10.1126/science.370.6519.895. PMID: 33214256.
20. Palacio N, Dangi T, Chung YR, Wang Y, Loredo-Varela JL, Zhang Z, Penaloza-MacMaster P. Early type I IFN blockade improves the efficacy of viral vaccines. J Exp Med. 2020 Dec 7;217(12):e20191220. doi: 10.1084/jem.20191220. PMID: 32820330.
21. Bosco-Lauth AM, Hartwig AE, Porter SM, Gordy PW, Nehring M, Byas AD, VandeWoude S, Ragan IK, Maison RM, Bowen RA. Experimental infection of domestic dogs and cats with SARS-CoV-2: Pathogenesis, transmission, and response to reexposure in cats. Proc Natl Acad Sci U S A. 2020 Oct 20;117(42):26382-26388. doi: 10.1073/pnas.2013102117. Epub 2020 Sep 29. PMID: 32994343; PMCID: PMC7585007.
22. He S, Han J, Lichtfouse E. Backward transmission of COVID-19 from humans to animals may propagate reinfections and induce vaccine failure. Environ Chem Lett. 2021 Jan 3:1-6. doi: 10.1007/s10311-020-01140-4. Epub ahead of print. PMID: 33424524; PMCID: PMC7779092.
23. Zarletti G, Tiberi M, De Molfetta V, Bossù M, Toppi E, Bossù P, Scapigliati G. A Cell-Based ELISA to Improve the Serological Analysis of Anti-SARS-CoV-2 IgG. Viruses. 2020 Nov 8;12(11):1274. doi: 10.3390/v12111274. PMID: 33171590; PMCID: PMC7695166.
24. Muecksch F, Wise H, Batchelor B, Squires M, Semple E, Richardson C, McGuire J, Clearly S, Furrie E, Greig N, Hay G, Templeton K, Lorenzi JCC, Hatziioannou T, Jenks S, Bieniasz PD. Longitudinal analysis of serology and neutralizing antibody levels in COVID19 convalescents. J Infect Dis. 2020 Nov 3:jiaa659. doi: 10.1093/infdis/jiaa659. Epub ahead of print. PMID: 33140086; PMCID: PMC7665595.
25. Ibrahim M, Vegel A, Niu A, Panse K, Chen R, Safah H, Socola F, Luk A, Saba NS. Reinfection versus failure of viral clearance in a COVID-19 patient with hematologic malignancy. Leuk Res. 2021 Jan 20;101:106514. doi: 10.1016/j.leukres.2021.106514. Epub ahead of print. PMID: 33524639; PMCID: PMC7817416.
26. Patwardhan A. Sustained Positivity and Reinfection With SARS-CoV-2 in Children: Does Quarantine/Isolation Period Need Reconsideration in a Pediatric Population? Cureus. 2020 Dec 10;12(12):e12012. doi: 10.7759/cureus.12012. PMID: 33324532; PMCID: PMC7732141.
27. Bose S, Adapa S, Konala VM, Gopalreddy H, Sohail S, Naramala S, Kondakindi H, Muppidi V, Ramachandran MV, Juran PJ, Aeddula NR. Atypical Presentation of Novel Coronavirus Disease 2019 in a Peritoneal Dialysis Patient. J Investig Med High Impact Case Rep. 2020 Jan-Dec;8:2324709620931238. doi: 10.1177/2324709620931238. PMID: 32525402; PMCID: PMC7290252.
28. Dos Santos WG. Impact of virus genetic variability and host immunity for the success of COVID-19 vaccines. Biomed Pharmacother. 2021 Jan 12;136:111272. doi: 10.1016/j.biopha.2021.111272. Epub ahead of print. PMID: 33486212; PMCID: PMC7802525.
29. Mayne ES, Scott L, Semete B, Julsing A, Jugwanth S, Mampeule N, David A, Gededzha MP, Goga A, Hardie D, Preiser W, Chetty K, Rees H, Sanne I, Mlisana K, George JA, Stevens W. The role of serological testing in the SARS-CoV-2 outbreak. S Afr Med J. 2020 Jul 17;110(9):842-845. PMID: 32880264.
30. Addetia A, Crawford KHD, Dingens A, Zhu H, Roychoudhury P, Huang ML, Jerome KR, Bloom JD, Greninger AL. Neutralizing Antibodies Correlate with Protection from SARS-CoV-2 in Humans during a Fishery Vessel Outbreak with a High Attack Rate. J Clin Microbiol. 2020 Oct 21;58(11):e02107-20. doi: 10.1128/JCM.02107-20. PMID: 32826322; PMCID: PMC7587101.
31. Nag DS, Chaudhry R, Mishra M, Rai S, Gupta M. A Prospective Study on Rapidly Declining SARS-CoV-2 IgG Antibodies Within One to Three Months of Testing IgG Positive: Can It Lead to Potential Reinfections? Cureus. 2020 Dec 2;12(12):e11845. doi: 10.7759/cureus.11845. PMID: 33282604; PMCID: PMC7714733.

EXCLI Journal 2021;20:522-536 – ISSN 1611-2156
Received: January 15, 2021, accepted: February 23, 2021, published: March 02, 2021

32. Sanclemente-Alaman I, Moreno-Jiménez L, Benito-Martín MS, Canales-Aguirre A, Matías-Guiu JA, Matías-Guiu J, Gómez-Pinedo U. Experimental Models for the Study of Central Nervous System Infection by SARS-CoV-2. Front Immunol. 2020 Aug 28;11:2163. doi: 10.3389/fimmu.2020.02163. PMID: 32983181; PMCID: PMC7485091.
33. Moore JL, Ganapathiraju PV, Kurtz CP, Wainscoat B. A 63-Year-Old Woman with a History of Non-Hodgkin Lymphoma with Persistent SARS-CoV-2 Infection Who Was Seronegative and Treated with Convalescent Plasma. Am J Case Rep. 2020 Oct 3;21:e927812. doi: 10.12659/AJCR.927812. PMID: 33009361; PMCID: PMC7542548.
34. Gryseels S, De Bruyn L, Gyselings R, Calvignac-Spencer S, Leendertz FH, Leirs H. Risk of human-to-wildlife transmission of SARS-CoV-2. Mamm Rev. 2020 Oct 6:10.1111/mam.12225. doi: 10.1111/mam.12225. Epub ahead of print. PMID: 33230363; PMCID: PMC7675675.
35. Alhusseini LB, Yassen LT, Kouhsari E, Al Marjani MF. Persistence of SARS-CoV-2: a new paradigm of COVID-19 management. Ann Ig. 2021 Jan 14. doi: 10.7416/ai.2021.2414. Epub ahead of print. PMID: 33443283.
36. Núñez-López M, Alarcón Ramos L, Velasco-Hernández JX. Migration rate estimation in an epidemic network. Appl Math Model. 2021 Jan;89:1949-1964. doi: 10.1016/j.apm.2020.08.025. Epub 2020 Sep 12. PMID: 32952269; PMCID: PMC7486824.
37. Tillett RL, Sevinsky JR, Hartley PD, Kerwin H, Crawford N, Gorzalski A, Laverdure C, Verma SC, Rossetto CC, Jackson D, Farrell MJ, Van Hooser S, Pandori M. Genomic evidence for reinfection with SARS-CoV-2: a case study. Lancet Infect Dis. 2021 Jan;21(1):52-58. doi: 10.1016/S1473-3099(20)30764-7. Epub 2020 Oct 12. PMID: 33058797; PMCID: PMC7550103.
38. Huang J, Zheng L, Li Z, Hao S, Ye F, Chen J, Gans HA, Yao X, Liao J, Wang S, Zeng M, Qiu L, Li C, Whitin JC, Tian L, Chubb H, Hwa KY, Ceresnak SR, Zhang W, Lu Y, Maldonado YA, McElhinney DB, Sylvester KG, Cohen HJ, Liu L, Ling XB. Kinetics of SARS-CoV-2 positivity of infected and recovered patients from a single center. Sci Rep. 2020 Oct 29;10(1):18629. doi: 10.1038/s41598-020-75629-x. PMID: 33122706; PMCID: PMC7596704.
39. Florea AA, Sirbu CA, Ghinescu MC, Plesa CF, Sirbu AM, Mitrica M, Ionita-Radu F. SARS-CoV-2, multiple sclerosis, and focal deficit in a postpartum woman: A case report. Exp Ther Med. 2021 Jan;21(1):92. doi: 10.3892/etm.2020.9524. Epub 2020 Nov 26. PMID: 33363603; PMCID: PMC7725028.
40. Prado-Vivar B, Becerra-Wong M, Guadalupe JJ, Márquez S, Gutierrez B, Rojas-Silva P, Grunauer M, Trueba G, Barragán V, Cárdenas P. A case of SARS-CoV-2 reinfection in Ecuador. Lancet Infect Dis. 2020 Nov 23:S1473-3099(20)30910-5. doi: 10.1016/S1473-3099(20)30910-5. Epub ahead of print. PMID: 33242475; PMCID: PMC7833993.
41. Sette A, Crotty S. Adaptive immunity to SARS-CoV-2 and COVID-19. Cell. 2021 Jan 12:S0092-8674(21)00007-6. doi: 10.1016/j.cell.2021.01.007. Epub ahead of print. PMID: 33497610; PMCID: PMC7803150.
42. Ozaras R, Ozdogru I, Yilmaz AA. Coronavirus disease 2019 re-infection: first report from Turkey. New Microbes New Infect. 2020 Oct 3;38:100774. doi: 10.1016/j.nmni.2020.100774. PMID: 33235800; PMCID: PMC7670194.
43. Tuan J, Spichler-Moffarah A, Ogbuagu O. A new positive SARS-CoV-2 test months after severe COVID-19 illness: reinfection or intermittent viral shedding? BMJ Case Rep. 2021 Feb 4;14(2):e240531. doi: 10.1136/bcr-2020-240531. PMID: 33542020.
44. Kim YI, Kim SM, Park SJ, Kim EH, Yu KM, Chang JH, Kim EJ, Casel MAB, Rollon R, Jang SG, Um J, Song MS, Jeong HW, Kim EG, Kim Y, Kim SY, Park JS, Park MS, Kwon GY, Yeo SG, Lee SA, Choi YJ, Jung JU, Choi YK. Critical role of neutralizing antibody for SARS-CoV-2 reinfection and transmission. Emerg Microbes Infect. 2021 Dec;10(1):152-160. doi: 10.1080/22221751.2021.1872352. PMID: 33407005; PMCID: PMC7832474.
45. Mustapha JO, Abdullahi IN, Ajagbe OOR, Emeribe AU, Fasogbon SA, Onoja SO, Ugwu CE, Umeozuru CM, Ajayi FO, Tanko WN, Omosigho PO, Aliyu AS, Shuwa HA, Nwofe JO, Dangana A, Alaba O, Ghamba PE, Ibrahim Y, Aliyu D, Animasaun OS, Ugboaja NB, Baba Mallam MA, Abubakar SD, Aminu MS, Yahaya H, Oyewusi S. Understanding the implications of SARS-CoV-2 re-infections on immune response milieu, laboratory tests and control measures against COVID-19. Heliyon. 2021 Jan 9;7(1):e05951. doi: 10.1016/j.heliyon.2021.e05951. PMID: 33490695; PMCID: PMC7810769.

46. Mahallawi W. Case Report: A Recovered SARS CoV-2 Patient Protected From Reinfection. Front Med (Lausanne). 2020 Sep 25;7:564264. doi: 10.3389/fmed.2020.564264. PMID: 33102502; PMCID: PMC7544991.
47. Brochot E, Demey B, Touzé A, Belouzard S, Dubuisson J, Schmit JL, Duverlie G, Francois C, Castelain S, Helle F. Anti-spike, Anti-nucleocapsid and Neutralizing Antibodies in SARS-CoV-2 Inpatients and Asymptomatic Individuals. Front Microbiol. 2020 Oct 19;11:584251. doi: 10.3389/fmicb.2020.584251. PMID: 33193227; PMCID: PMC7604306.
48. Yamamoto H, Miura K, Hagiwara K, Morisawa Y, Lefor AK, Sata N, Nagai R. A Strategy to Battle Coronavirus Disease 2019 in the Hospital: Identify and Use the Power of "Immune Survivors". JMA J. 2020 Oct 15;3(4):303-306. doi: 10.31662/jmaj.2020-0039. Epub 2020 Oct 2. PMID: 33225101; PMCID: PMC7676985.
49. Carrillo J, Izquierdo-Useros N, Ávila-Nieto C, Pradenas E, Clotet B, Blanco J. Humoral immune responses and neutralizing antibodies against SARS-CoV-2; implications in pathogenesis and protective immunity. Biochem Biophys Res Commun. 2020 Nov 7:S0006-291X(20)32037-4. doi: 10.1016/j.bbrc.2020.10.108. Epub ahead of print. PMID: 33187644; PMCID: PMC7648524.
50. Quast I, Tarlinton D. B cell memory: understanding COVID-19. Immunity. 2021 Jan 23:S1074-7613(21)00037-6. doi: 10.1016/j.immuni.2021.01.014. Epub ahead of print. PMID: 33513337; PMCID: PMC7826135.
51. Novoa W, Miller H, Mattar S, Faccini-Martínez ÁA, Rivero R, Serrano-Coll H. A first probable case of SARS-CoV-2 reinfection in Colombia. Ann Clin Microbiol Antimicrob. 2021 Jan 12;20(1):7. doi: 10.1186/s12941-020-00413-8. PMID: 33435982; PMCID: PMC7802059.
52. Lumley SF, Wei J, O'Donnell D, Stoesser NE, Matthews PC, Howarth A, Hatch SB, Marsden BD, Cox S, James T, Peck LJ, Ritter TG, de Toledo Z, Cornall RJ, Jones EY, Stuart DI, Screaton G, Ebner D, Hoosdally S, Crook DW, Conlon CP, Pouwels KB, Walker AS, Peto TEA, Walker TM, Jeffery K, Eyre DW; Oxford University Hospitals Staff Testing Group. The duration, dynamics and determinants of SARS-CoV-2 antibody responses in individual healthcare workers. Clin Infect Dis. 2021 Jan 6:ciab004. doi: 10.1093/cid/ciab004. Epub ahead of print. PMID: 33400782.
53. Shayak B, Sharma MM, Gaur M, Mishra AK. Impact of reproduction number on multiwave spreading dynamics of COVID-19 with temporary immunity: a mathematical model. Int J Infect Dis. 2021 Jan 11:S1201-9712(21)00028-X. doi: 10.1016/j.ijid.2021.01.018. Epub ahead of print. PMID: 33444746; PMCID: PMC7836705.
54. Mohapatra S, Menon NG, Mohapatra G, Pisharody L, Pattnaik A, Menon NG, Bhukya PL, Srivastava M, Singh M, Barman MK, Gin KY, Mukherji S. The novel SARS-CoV-2 pandemic: Possible environmental transmission, detection, persistence and fate during wastewater and water treatment. Sci Total Environ. 2020 Oct 6:142746. doi: 10.1016/j.scitotenv.2020.142746. Epub ahead of print. PMID: 33092831; PMCID: PMC7536135.
55. Fong B, London KS. Prolonged Duration of Viral Shedding of SARS-CoV-2: A Case Report. Clin Pract Cases Emerg Med. 2020 Nov;4(4):509-512. doi: 10.5811/cpcem.2020.7.49005. PMID: 33217258; PMCID: PMC7676783.
56. Boonyaratanakornkit J, Morishima C, Selke S, Zamora D, McGuffin S, Shapiro AE, Campbell VL, McClurkan CL, Jing L, Gross R, Liang J, Postnikova E, Mazur S, Lukin VV, Chaudhary A, Das MK, Fink SL, Bryan A, Greninger AL, Jerome KR, Holbrook MR, Gernsheimer TB, Wener MH, Wald A, Koelle DM. Clinical, laboratory, and temporal predictors of neutralizing antibodies against SARS-CoV-2 among COVID-19 convalescent plasma donor candidates. J Clin Invest. 2021 Feb 1;131(3):e144930. doi: 10.1172/JCI144930. PMID: 33320842; PMCID: PMC7843229.
57. Kahn R, Kennedy-Shaffer L, Grad YH, Robins JM, Lipsitch M. Potential Biases Arising from Epidemic Dynamics in Observational Seroprotection Studies. Am J Epidemiol. 2020 Sep 1;190(2):328–35. doi: 10.1093/aje/kwaa188. Epub ahead of print. PMID: 32870977; PMCID: PMC7499481.
58. van der Hoek L. SARS-CoV-2 Re-infections: Lessons from Other Coronaviruses. Med (N Y). 2020 Dec 18;1(1):23-28. doi: 10.1016/j.medj.2020.12.005. PMID: 33521751; PMCID: PMC7836792.
59. Almansour I, Macadato NC, Alshammari T. Immunogenicity of Multiple Doses of pDNA Vaccines against SARS-CoV-2. Pharmaceuticals (Basel). 2021 Jan 6;14(1):39. doi: 10.3390/ph14010039. PMID: 33419184; PMCID: PMC7825551.

60. Grimwood K, Lambert SB, Ware RS. Endemic Non-SARS-CoV-2 Human Coronaviruses in a Community-Based Australian Birth Cohort. Pediatrics. 2020 Nov;146(5):e2020009316. doi: 10.1542/peds.2020-009316. Epub 2020 Sep 4. PMID: 32887791.
61. Zhang J, Xu J, Zhou S, Wang C, Wang X, Zhang W, Ning K, Pan Y, Liu T, Zhao J, Dong L. The characteristics of 527 discharged COVID-19 patients undergoing long-term follow-up in China. Int J Infect Dis. 2021 Feb 1:S1201-9712(21)00075-8. doi: 10.1016/j.ijid.2021.01.064. Epub ahead of print. PMID: 33540129; PMCID: PMC7848496.
62. Salcin SAMEENA, Fontem FRANKLIN. RECURRENT SARS-CoV-2 INFECTION RESULTING IN ACUTE RESPIRATORY DISTRESS SYNDROME AND DEVELOPMENT OF PULMONARY HYPERTENSION: A CASE REPORT. Respir Med Case Rep. 2020 Dec 5:101314. doi: 10.1016/j.rmcr.2020.101314. Epub ahead of print. PMID: 33312856; PMCID: PMC7718582.
63. Lechien JR, Chiesa-Estomba CM, Radulesco T, Michel J, Varia LA, Le Bon SD, Horoi M, Falanga C, Barillari MR, Hans S, Tucciarone M, Saussez S. Clinical Features of Patients who had two COVID-19 Episodes: A European Multicenter Case-series. J Intern Med. 2021 Feb 1. doi: 10.1111/joim.13259. Epub ahead of print. PMID: 33527495.
64. Abu-Raddad LJ, Chemaitelly H, Malek JA, Ahmed AA, Mohamoud YA, Younuskunju S, Al Kanaani Z, Al Khal A, Al Kuwari E, Butt AA, Coyle P, Jeremijenko A, Kaleeckal AH, Latif AN, Shaik RM, Abdul Rahim HF, Yassine HM, Al Kuwari MG, Al Romaihi HE, Al-Thani MH, Bertollini R. Two prolonged viremic SARS-CoV-2 infections with conserved viral genome for two months. Infect Genet Evol. 2020 Dec 24;88:104684. doi: 10.1016/j.meegid.2020.104684. Epub ahead of print. PMID: 33352320; PMCID: PMC7759339.
65. Addie D, Houe L, Maitland K, Passantino G, Decaro N. Effect of cat litters on feline coronavirus infection of cell culture and cats. J Feline Med Surg. 2020 Apr;22(4):350-357. doi: 10.1177/1098612X19848167. Epub 2019 May 16. PMID: 31094626.
66. Aljasim TA, Almasoud A, Aljami HA, Alenazi MW, Alsagaby SA, Alsaleh AN, Alharbi NK. High Rate of Circulating MERS-CoV in Dromedary Camels at Slaughterhouses in Riyadh, 2019. Viruses. 2020 Oct 27;12(11):1215. doi: 10.3390/v12111215. PMID: 33120981; PMCID: PMC7692456.
67. Oved K, Olmer L, Shemer-Avni Y, Wolf T, Supino-Rosin L, Prajgrod G, Shenhar Y, Payorsky I, Cohen Y, Kohn Y, Indenbaum V, Lazar R, Geylis V, Oikawa MT, Shinar E, Stoyanov E, Keinan-Boker L, Bassal R, Reicher S, Yishai R, Bar-Chaim A, Doolman R, Reiter Y, Mendelson E, Livneh Z, Freedman LS, Lustig Y. Multi-center nationwide comparison of seven serology assays reveals a SARS-CoV-2 non-responding seronegative subpopulation. EClinicalMedicine. 2020 Dec;29:100651. doi: 10.1016/j.eclinm.2020.100651. Epub 2020 Nov 19. PMID: 33235985; PMCID: PMC7676374.
68. Guthmiller JJ, Stovicek O, Wang J, Changrob S, Li L, Halfmann P, Zheng NY, Utset H, Stamper CT, Dugan HL, Miller WD, Huang M, Dai YN, Nelson CA, Hall PD, Jansen M, Shanmugarajah K, Donington JS, Krammer F, Fremont DH, Joachimiak A, Kawaoka Y, Tesic V, Madariaga ML, Wilson PC. SARS-CoV-2 Infection Severity Is Linked to Superior Humoral Immunity against the Spike. mBio. 2021 Jan 19;12(1):e02940-20. doi: 10.1128/mBio.02940-20. PMID: 33468695.
69. Turner JS, Day A, Alsoussi WB, Liu Z, O'Halloran JA, Presti RM, Patterson BK, Whelan SPJ, Ellebedy AH, Mudd PA. SARS-CoV-2 Viral RNA Shedding for More Than 87 Days in an Individual With an Impaired CD8+ T Cell Response. Front Immunol. 2021 Jan 8;11:618402. doi: 10.3389/fimmu.2020.618402. PMID: 33488630; PMCID: PMC7820941.
70. Mandal S, Das H, Deo S, Arinaminpathy N. Combining serology with case-detection, to allow the easing of restrictions against SARS-CoV-2: a modelling-based study in India. Sci Rep. 2021 Jan 19;11(1):1835. doi: 10.1038/s41598-021-81405-2. PMID: 33469083; PMCID: PMC7815778.
71. Addetia A, Crawford KH, Dingens A, Zhu H, Roychoudhury P, Huang ML, Jerome KR, Bloom JD, Greninger AL. Neutralizing antibodies correlate with protection from SARS-CoV-2 in humans during a fishery vessel outbreak with high attack rate. medRxiv [Preprint]. 2020 Aug 14:2020.08.13.20173161. doi: 10.1101/2020.08.13.20173161. Update in: J Clin Microbiol. 2020 Aug 21;: PMID: 32817980; PMCID: PMC7430625.
72. Wibmer CK, Ayres F, Hermanus T, Madzivhandila M, Kgagudi P, Lambson BE, Vermeulen M, van den Berg K, Rossouw T, Boswell M, Ueckermann V, Meiring S, von Gottberg A, Cohen C, Morris L, Bhiman JN, Moore PL. SARS-CoV-2 501Y.V2 escapes neutralization by South African COVID-19 donor plasma. bioRxiv [Preprint]. 2021 Jan 19:2021.01.18.427166. doi: 10.1101/2021.01.18.427166. PMID: 33501446; PMCID: PMC7836116.

73. Goss MB, Moreno NF, Galván NTN, Rana A. Non-contagious, second COVID-19 infection: Implications for organ donation eligibility. Clin Transplant. 2020 Sep;34(9):e14039. doi: 10.1111/ctr.14039. Epub 2020 Aug 2. PMID: 32645230; PMCID: PMC7361207.
74. Osman AA, Al Daajani MM, Alsahafi AJ. Re-positive coronavirus disease 2019 PCR test: could it be a reinfection? New Microbes New Infect. 2020 Sep;37:100748. doi: 10.1016/j.nmni.2020.100748. Epub 2020 Aug 20. PMID: 32843984; PMCID: PMC7439804.
75. Azam M, Sulistiana R, Ratnawati M, Fibriana AI, Bahrudin U, Widyaningrum D, Aljunid SM. Recurrent SARS-CoV-2 RNA positivity after COVID-19: a systematic review and meta-analysis. Sci Rep. 2020 Nov 26;10(1):20692. doi: 10.1038/s41598-020-77739-y. PMID: 33244060; PMCID: PMC7691365.
76. de Brito CAA, Lima PMA, de Brito MCM, de Oliveira DB. Second Episode of COVID-19 in Health Professionals: Report of Two Cases. Int Med Case Rep J. 2020 Oct 2;13:471-475. doi: 10.2147/IMCRJ.S277882. PMID: 33061670; PMCID: PMC7537988.
77. Mahajan A, Manchikanti L. Value and Validity of Coronavirus Antibody Testing. Pain Physician. 2020 Aug;23(4S):S381-S390. PMID: 32942795.
78. Li Q, Zheng XS, Shen XR, Si HR, Wang X, Wang Q, Li B, Zhang W, Zhu Y, Jiang RD, Zhao K, Wang H, Shi ZL, Zhang HL, Du RH, Zhou P. Prolonged shedding of severe acute respiratory syndrome coronavirus 2 in patients with COVID-19. Emerg Microbes Infect. 2020 Dec;9(1):2571-2577. doi: 10.1080/22221751.2020.1852058. PMID: 33196399; PMCID: PMC7734137.
79. Alvarez-Moreno CA, Rodríguez-Morales AJ. Testing Dilemmas: Post negative, positive SARS-CoV-2 RT-PCR - is it a reinfection? Travel Med Infect Dis. 2020 May-Jun;35:101743. doi: 10.1016/j.tmaid.2020.101743. Epub 2020 May 15. PMID: 32416153; PMCID: PMC7228728.
80. Yang C, Jiang M, Wang X, Tang X, Fang S, Li H, Zuo L, Jiang Y, Zhong Y, Chen Q, Zheng C, Wang L, Wu S, Wu W, Liu H, Yuan J, Liao X, Zhang Z, Shi X, Geng Y, Zhang H, Zheng H, Wan M, Lu L, Ren X, Cui Y, Zou X, Feng T, Xia J, Yang R, Liu Y, Mei S, Li B, Yang Z, Hu Q. Viral RNA level, serum antibody responses, and transmission risk in recovered COVID-19 patients with recurrent positive SARS-CoV-2 RNA test results: a population-based observational cohort study. Emerg Microbes Infect. 2020 Dec;9(1):2368-2378. doi: 10.1080/22221751.2020.1837018. PMID: 33151135; PMCID: PMC7655076.
81. Khoshkam Z, Aftabi Y, Stenvinkel P, Paige Lawrence B, Rezaei MH, Ichihara G, Fereidouni S. Recovery scenario and immunity in COVID-19 disease: A new strategy to predict the potential of reinfection. J Adv Res. 2021 Jan 5. doi: 10.1016/j.jare.2020.12.013. Epub ahead of print. PMID: 33520309; PMCID: PMC7832464.
82. Zheng J, Zhou R, Chen F, Tang G, Wu K, Li F, Liu H, Lu J, Zhou J, Yang Z, Yuan Y, Lei C, Wu X. Incidence, clinical course and risk factor for recurrent PCR positivity in discharged COVID-19 patients in Guangzhou, China: A prospective cohort study. PLoS Negl Trop Dis. 2020 Aug 31;14(8):e0008648. doi: 10.1371/journal.pntd.0008648. PMID: 32866168; PMCID: PMC7505432.
83. Goldman JD, Wang K, Roltgen K, Nielsen SCA, Roach JC, Naccache SN, Yang F, Wirz OF, Yost KE, Lee JY, Chun K, Wrin T, Petropoulos CJ, Lee I, Fallen S, Manner PM, Wallick JA, Algren HA, Murray KM, Su Y, Hadlock J, Jeharajah J, Berrington WR, Pappas GP, Nyatsatsang ST, Greninger AL, Satpathy AT, Pauk JS, Boyd SD, Heath JR. Reinfection with SARS-CoV-2 and Failure of Humoral Immunity: a case report. medRxiv [Preprint]. 2020 Sep 25:2020.09.22.20192443. doi: 10.1101/2020.09.22.20192443. PMID: 32995830; PMCID: PMC7523175.
84. Lu L, Zhang H, Zhan M, Jiang J, Yin H, Dauphars DJ, Li SY, Li Y, He YW. Antibody response and therapy in COVID-19 patients: what can be learned for vaccine development? Sci China Life Sci. 2020 Dec;63(12):1833-1849. doi: 10.1007/s11427-020-1859-y. Epub 2020 Dec 1. PMID: 33355886; PMCID: PMC7756132.
85. Ledford H. Coronavirus reinfections: three questions scientists are asking. Nature. 2020 Sep;585(7824):168-169. doi: 10.1038/d41586-020-02506-y. PMID: 32887957.
86. Ueffing M, Bayyoud T, Schindler M, Ziemssen F. Grundlagen der Replikation und der Immunologie von SARS-CoV-2 [Basic principles of replication and immunology of SARS-CoV-2]. Ophthalmologe. 2020 Jul;117(7):609-614. German. doi: 10.1007/s00347-020-01155-w. Erratum in: Ophthalmologe. 2020 Aug;117(8):828. PMID: 32613257; PMCID: PMC7328300.
87. Overbaugh J. Understanding protection from SARS-CoV-2 by studying reinfection. Nat Med. 2020 Nov;26(11):1680-1681. doi: 10.1038/s41591-020-1121-z. PMID: 33093682.
88. Cohen JI, Burbelo PD. Reinfection with SARS-CoV-2: Implications for Vaccines. Clin Infect Dis. 2020 Dec 18:ciaa1866. doi: 10.1093/cid/ciaa1866. Epub ahead of print. PMID: 33338197; PMCID: PMC7799323.

89. Gaebler C, Wang Z, Lorenzi JCC, Muecksch F, Finkin S, Tokuyama M, Cho A, Jankovic M, Schaefer-Babajew D, Oliveira TY, Cipolla M, Viant C, Barnes CO, Hurley A, Turroja M, Gordon K, Millard KG, Ramos V, Schmidt F, Weisblum Y, Jha D, Tankelevich M, Yee J, Shimeliovich I, Robbiani DF, Zhao Z, Gazumyan A, Hatziioannou T, Bjorkman PJ, Mehandru S, Bieniasz PD, Caskey M, Nussenzweig MC, Hagglof T, Schwartz RE, Bram Y, Martinez-Delgado G, Mendoza P, Breton G, Dizon J, Unson-O'Brien C, Patel R. Evolution of Antibody Immunity to SARS-CoV-2. bioRxiv [Preprint]. 2020 Nov 5:2020.11.03.367391. doi: 10.1101/2020.11.03.367391. Update in: Nature. 2021 Jan 18;: PMID: 33173867; PMCID: PMC7654855.
90. Fernandes Valente Takeda C, Moura de Almeida M, Gonçalves de Aguiar Gomes R, Cisne Souza T, Alves de Lima Mota M, Pamplona de Góes Cavalcanti L, Baima Colares JK. Case Report: Recurrent Clinical Symptoms of COVID-19 in Healthcare Professionals: A Series of Cases from Brazil. Am J Trop Med Hyg. 2020 Nov;103(5):1993-1996. doi: 10.4269/ajtmh.20-0893. PMID: 32888288; PMCID: PMC7646791.
91. Ghali A, Rico-Mesa JS, Nashawi M, Cadena J. Cases and etiologies of suspected COVID-19 reactivation. Qatar Med J. 2020 Oct 13;2020(2):26. doi: 10.5339/qmj.2020.26. PMID: 33282710; PMCID: PMC7684550.
92. Wang QX, Huang KC, Qi L, Zeng XH, Zheng SL. No infectious risk of COVID-19 patients with long-term fecal 2019-nCoV nucleic acid positive. Eur Rev Med Pharmacol Sci. 2020 May;24(10):5772-5777. doi: 10.26355/eurrev_202005_21370. PMID: 32495914.
93. Kim DS, Rowland-Jones S, Gea-Mallorquí E. Will SARS-CoV-2 Infection Elicit Long-Lasting Protective or Sterilising Immunity? Implications for Vaccine Strategies (2020). Front Immunol. 2020 Dec 9;11:571481. doi: 10.3389/fimmu.2020.571481. PMID: 33362759; PMCID: PMC7756008.
94. Kalkeri R, Goebel S, Sharma GD. SARS-CoV-2 Shedding from Asymptomatic Patients: Contribution of Potential Extrapulmonary Tissue Reservoirs. Am J Trop Med Hyg. 2020 Jul;103(1):18-21. doi: 10.4269/ajtmh.20-0279. Epub 2020 May 13. PMID: 32406369; PMCID: PMC7356473.
95. Galipeau Y, Greig M, Liu G, Driedger M, Langlois MA. Humoral Responses and Serological Assays in SARS-CoV-2 Infections. Front Immunol. 2020 Dec 18;11:610688. doi: 10.3389/fimmu.2020.610688. PMID: 33391281; PMCID: PMC7775512.
96. Henriksen S, Trydal Ø, Sigurdsen SE, Tylden GD, Rinaldo CH. Antibody response in a family with COVID-19. Tidsskr Nor Laegeforen. 2020 Jul 23;140(11). English, Norwegian. doi: 10.4045/tidsskr.20.0420. PMID: 32815342.
97. Stadlbauer D, Amanat F, Chromikova V, Jiang K, Strohmeier S, Arunkumar GA, Tan J, Bhavsar D, Capuano C, Kirkpatrick E, Meade P, Brito RN, Teo C, McMahon M, Simon V, Krammer F. SARS-CoV-2 Seroconversion in Humans: A Detailed Protocol for a Serological Assay, Antigen Production, and Test Setup. Curr Protoc Microbiol. 2020 Jun;57(1):e100. doi: 10.1002/cpmc.100. PMID: 32302069; PMCID: PMC7235504
98. Krsak M, Henao-Martínez AF, Franco-Paredes C. COVID-19: Way Forward With Serosurveillance Without Overemphasizing Neutralizing Antibodies. Viral Immunol. 2020 Nov 11. doi: 10.1089/vim.2020.0246. Epub ahead of print. PMID: 33181055.
99. Zhao G, Su Y, Sun X, Cui X, Dang L, Zhao L, Tan X, Wang H, Yang M. A comparative study of the laboratory features of COVID-19 and other viral pneumonias in the recovery stage. J Clin Lab Anal. 2020 Oct;34(10):e23483. doi: 10.1002/jcla.23483. Epub 2020 Jul 21. PMID: 32696465; PMCID: PMC7404602.
100. Bruni M, Cecatiello V, Diaz-Basabe A, Lattanzi G, Mileti E, Monzani S, Pirovano L, Rizzelli F, Visintin C, Bonizzi G, Giani M, Lavitrano M, Faravelli S, Forneris F, Caprioli F, Pelicci PG, Natoli G, Pasqualato S, Mapelli M, Facciotti F. Persistence of Anti-SARS-CoV-2 Antibodies in Non-Hospitalized COVID-19 Convalescent Health Care Workers. J Clin Med. 2020 Oct 1;9(10):3188. doi: 10.3390/jcm9103188. PMID: 33019628; PMCID: PMC7600936.
101. Bellesso M, Bruniera FR, Trunkel AT, Nicodemo IP. Second COVID-19 infection in a patient with multiple myeloma in Brazil - reinfection or reactivation? Hematol Transfus Cell Ther. 2020 Dec 19:S2531-1379(20)31308-0. doi: 10.1016/j.htct.2020.12.002. Epub ahead of print. PMID: 33423984; PMCID: PMC7837121.
102. Van Elslande J, Vermeersch P, Vandervoort K, Wawina-Bokalanga T, Vanmechelen B, Wollants E, Laenen L, André E, Van Ranst M, Lagrou K, Maes P. Symptomatic SARS-CoV-2 reinfection by a phylogenetically distinct strain. Clin Infect Dis. 2020 Sep 5:ciaa1330. doi: 10.1093/cid/ciaa1330. Epub ahead of print. PMID: 32887979; PMCID: PMC7499557.
103. Pal R, Banerjee M. Are people with uncontrolled diabetes mellitus at high risk of reinfections with COVID-19? Prim Care Diabetes. 2021 Feb;15(1):18-20. doi: 10.1016/j.pcd.2020.08.002. Epub 2020 Aug 7. PMID: 32800450; PMCID: PMC7413202.

104. Daniel P, Raad M, Waked R, Choucair J, Riachy M, Haddad F. COVID-19 in a Patient Treated for Granulomatosis with Polyangiitis: Persistent Viral Shedding with No Cytokine Storm. Eur J Case Rep Intern Med. 2020 Sep 24;7(10):001922. doi: 10.12890/2020_001922. PMID: 33083371; PMCID: PMC7546569.
105. Seow J, Graham C, Merrick B, Acors S, Pickering S, Steel KJA, Hemmings O, O'Byrne A, Kouphou N, Galao RP, Betancor G, Wilson HD, Signell AW, Winstone H, Kerridge C, Huettner I, Jimenez-Guardeño JM, Lista MJ, Temperton N, Snell LB, Bisnauthsing K, Moore A, Green A, Martinez L, Stokes B, Honey J, Izquierdo-Barras A, Arbane G, Patel A, Tan MKI, O'Connell L, O'Hara G, MacMahon E, Douthwaite S, Nebbia G, Batra R, Martinez-Nunez R, Shankar-Hari M, Edgeworth JD, Neil SJD, Malim MH, Doores KJ. Longitudinal observation and decline of neutralizing antibody responses in the three months following SARS-CoV-2 infection in humans. Nat Microbiol. 2020 Dec;5(12):1598-1607. doi: 10.1038/s41564-020-00813-8. Epub 2020 Oct 26. PMID: 33106674.
106. Pavia CS, Wormser GP. COVID-19: Is there a role for Western blots and skin testing for determining immunity and development of a vaccine? Diagn Microbiol Infect Dis. 2020 Dec;98(4):115148. doi: 10.1016/j.diagmicrobio.2020.115148. Epub 2020 Aug 13. PMID: 32920452; PMCID: PMC7424330.
107. Voo TC, Clapham H, Tam CC. Ethical Implementation of Immunity Passports During the COVID-19 Pandemic. J Infect Dis. 2020 Aug 4;222(5):715-718. doi: 10.1093/infdis/jiaa352. PMID: 32582943; PMCID: PMC7337820.
108. Hausburg MA, Banton KL, Roshon M, Bar-Or D. Clinically distinct COVID-19 cases share notably similar immune response progression: A follow-up analysis. Heliyon. 2020 Dec 31;7(1):e05877. doi: 10.1016/j.heliyon.2020.e05877. PMID: 33437888; PMCID: PMC7788102.
109. Breathnach AS, Riley PA, Cotter MP, Houston AC, Habibi MS, Planche TD. Prior COVID-19 significantly reduces the risk of subsequent infection, but reinfections are seen after eight months. J Infect. 2021 Jan 13:S0163-4453(21)00010-4. doi: 10.1016/j.jinf.2021.01.005. Epub ahead of print. PMID: 33450303; PMCID: PMC7804382.
110. Woolsey C, Borisevich V, Prasad AN, Agans KN, Deer DJ, Dobias NS, Heymann JC, Foster SL, Levine CB, Medina L, Melody K, Geisbert JB, Fenton KA, Geisbert TW, Cross RW. Establishment of an African green monkey model for COVID-19 and protection against re-infection. Nat Immunol. 2021 Jan;22(1):86-98. doi: 10.1038/s41590-020-00835-8. Epub 2020 Nov 24. PMID: 33235385; PMCID: PMC7790436.
111. Habel JR, Nguyen THO, van de Sandt CE, Juno JA, Chaurasia P, Wragg K, Koutsakos M, Hensen L, Jia X, Chua B, Zhang W, Tan HX, Flanagan KL, Doolan DL, Torresi J, Chen W, Wakim LM, Cheng AC, Doherty PC, Petersen J, Rossjohn J, Wheatley AK, Kent SJ, Rowntree LC, Kedzierska K. Suboptimal SARS-CoV-2-specific $CD8^+$ T cell response associated with the prominent HLA-A*02:01 phenotype. Proc Natl Acad Sci U S A. 2020 Sep 29;117(39):24384-24391. doi: 10.1073/pnas.2015486117. Epub 2020 Sep 10. PMID: 32913053; PMCID: PMC7533701.
112. Tehrani HA, Darnahal M, Nadji SA, Haghighi S. COVID-19 re-infection or persistent infection in patient with acute myeloid leukaemia M3: a mini review. New Microbes New Infect. 2020 Dec 5;39:100830. doi: 10.1016/j.nmni.2020.100830. PMID: 33425365; PMCID: PMC7777517.
113. Wajnberg A, Mansour M, Leven E, Bouvier NM, Patel G, Firpo-Betancourt A, Mendu R, Jhang J, Arinsburg S, Gitman M, Houldsworth J, Sordillo E, Paniz-Mondolfi A, Baine I, Simon V, Aberg J, Krammer F, Reich D, Cordon-Cardo C. Humoral response and PCR positivity in patients with COVID-19 in the New York City region, USA: an observational study. Lancet Microbe. 2020 Nov;1(7):e283-e289. doi: 10.1016/S2666-5247(20)30120-8. Epub 2020 Sep 25. PMID: 33015652; PMCID: PMC7518831.
114. Taefehshokr N, Taefehshokr S, Heit B. Mechanisms of Dysregulated Humoral and Cellular Immunity by SARS-CoV-2. Pathogens. 2020 Dec 8;9(12):1027. doi: 10.3390/pathogens9121027. PMID: 33302366; PMCID: PMC7762606.
115. Batistela CM, Correa DPF, Bueno ÁM, Piqueira JRC. SIRSi compartmental model for COVID-19 pandemic with immunity loss. Chaos Solitons Fractals. 2021 Jan;142:110388. doi: 10.1016/j.chaos.2020.110388. Epub 2020 Oct 29. PMID: 33162689; PMCID: PMC7598795.
116. Shen G, Yang G, Zeng ZY, Hu Y, Li Q, Liu ZG, Fu HY, Hu JY, Zhu P, Huang JH, Lu QQ, Shi SJ, He Y, Xie XB. [Preliminary study of the antibody level in confirmed patients with COVID-19 after discharge]. Zhonghua Yu Fang Yi Xue Za Zhi. 2020 Dec 6;54(12):1448-1452. Chinese. doi: 10.3760/cma.j.cn112150-20200603-00819. PMID: 33333665.
117. Khan ZS, Van Bussel F, Hussain F. A predictive model for Covid-19 spread - with application to eight US states and how to end the pandemic. Epidemiol Infect. 2020 Oct 8;148:e249. doi: 10.1017/S0950268820002423. PMID: 33028445; PMCID: PMC7588724.

118. la Fuente J, Gortázar C, Cabezas-Cruz A. Immunity to glycan α-Gal and possibilities for the control of COVID-19. Immunotherapy. 2021 Feb;13(3):185-188. doi: 10.2217/imt-2020-0247. Epub 2020 Dec 14. PMID: 33307805; PMCID: PMC7735019.
119. Amor S, Fernández Blanco L, Baker D. Innate immunity during SARS-CoV-2: evasion strategies and activation trigger hypoxia and vascular damage. Clin Exp Immunol. 2020 Nov;202(2):193-209. doi: 10.1111/cei.13523. Epub 2020 Oct 12. PMID: 32978971; PMCID: PMC7537271.
120. Burda Z. Modelling Excess Mortality in Covid-19-Like Epidemics. Entropy (Basel). 2020 Oct 30;22(11):E1236. doi: 10.3390/e22111236. PMID: 33287004.
121. Lozada-Requena I, Núñez Ponce C. COVID-19: respuesta inmune y perspectivas terapéuticas. Rev Peru Med Exp Salud Publica. 2020 Apr-Jun;37(2):312-319. Spanish. doi: 10.17843/rpmesp.2020.372.5490. Epub 2020 Aug 28. PMID: 32876223.
122. O Murchu E, Byrne P, Walsh KA, Carty PG, Connolly M, De Gascun C, Jordan K, Keoghan M, O'Brien KK, O'Neill M, Smith SM, Teljeur C, Ryan M, Harrington P. Immune response following infection with SARS-CoV-2 and other coronaviruses: A rapid review. Rev Med Virol. 2020 Sep 23:e2162. doi: 10.1002/rmv.2162. Epub ahead of print. PMID: 32964627; PMCID: PMC7536965.
123. Nguyen-Contant P, Embong AK, Kanagaiah P, Chaves FA, Yang H, Branche AR, Topham DJ, Sangster MY. S Protein-Reactive IgG and Memory B Cell Production after Human SARS-CoV-2 Infection Includes Broad Reactivity to the S2 Subunit. mBio. 2020 Sep 25;11(5):e01991-20. doi: 10.1128/mBio.01991-20. PMID: 32978311; PMCID: PMC7520599.
124. Chen W, Hu Z, Yi C, Chi Y, Xiong Q, Tan CW, Yi Y, Wang LF. An unusual COVID-19 case with over four months of viral shedding in the presence of low neutralizing antibodies: a case report. J Biomed Res. 2020 Sep 16;34(6):470-474. doi: 10.7555/JBR.34.20200099. PMID: 33243942; PMCID: PMC7718073.
125. West R, Kobokovich A, Connell N, Gronvall GK. COVID-19 Antibody Tests: A Valuable Public Health Tool with Limited Relevance to Individuals. Trends Microbiol. 2020 Nov 6:S0966-842X(20)30280-8. doi: 10.1016/j.tim.2020.11.002. Epub ahead of print. PMID: 33234439; PMCID: PMC7836413.
126. Larson D, Brodniak SL, Voegtly LJ, Cer RZ, Glang LA, Malagon FJ, Long KA, Potocki R, Smith DR, Lanteri C, Burgess T, Bishop-Lilly KA. A Case of Early Re-infection with SARS-CoV-2. Clin Infect Dis. 2020 Sep 19:ciaa1436. doi: 10.1093/cid/ciaa1436. Epub ahead of print. PMID: 32949240; PMCID: PMC7543357.
127. Sterlin D, Mathian A, Miyara M, Mohr A, Anna F, Claër L, Quentric P, Fadlallah J, Devilliers H, Ghillani P, Gunn C, Hockett R, Mudumba S, Guihot A, Luyt CE, Mayaux J, Beurton A, Fourati S, Bruel T, Schwartz O, Lacorte JM, Yssel H, Parizot C, Dorgham K, Charneau P, Amoura Z, Gorochov G. IgA dominates the early neutralizing antibody response to SARS-CoV-2. Sci Transl Med. 2021 Jan 20;13(577):eabd2223. doi: 10.1126/scitranslmed.abd2223. Epub 2020 Dec 7. PMID: 33288662; PMCID: PMC7857408.
128. Chan PKS, Lui G, Hachim A, Ko RLW, Boon SS, Li T, Kavian N, Luk F, Chen Z, Yau EM, Chan KH, Tsang CH, Cheng SMS, Chu DKW, Perera RAPM, Ho WCS, Yeung ACM, Chow C, Poon LLM, Valkenburg SA, Hui DSC, Peiris M. Serologic Responses in Healthy Adult with SARS-CoV-2 Reinfection, Hong Kong, August 2020. Emerg Infect Dis. 2020 Dec;26(12):3076-3078. doi: 10.3201/eid2612.203833. Epub 2020 Oct 22. PMID: 33089772; PMCID: PMC7706979.
129. Munoz Mendoza J, Alcaide ML. COVID-19 in a patient with end-stage renal disease on chronic in-center hemodialysis after evidence of SARS-CoV-2 IgG antibodies. Reinfection or inaccuracy of antibody testing. IDCases. 2020;22:e00943. doi: 10.1016/j.idcr.2020.e00943. Epub 2020 Sep 6. PMID: 32923364; PMCID: PMC7474827.
130. Lee JS, Kim SY, Kim TS, Hong KH, Ryoo NH, Lee J, Park JH, Cho SI, Kim MJ, Kim YG, Kim B, Shin HS, Oh HS, Seo MS, Gwon TR, Kim Y, Park JS, Chin BS, Park WB, Park SS, Seong MW. Evidence of Severe Acute Respiratory Syndrome Coronavirus 2 Reinfection After Recovery from Mild Coronavirus Disease 2019. Clin Infect Dis. 2020 Nov 21:ciaa1421. doi: 10.1093/cid/ciaa1421. Epub ahead of print. PMID: 33219681.
131. Amoozgar B, Kaushal V, Mubashar U, Sen S, Yousaf S, Yotsuya M. Symptomatic pericardial effusion in the setting of asymptomatic COVID-19 infection: A case report. Medicine (Baltimore). 2020 Sep 11;99(37):e22093. doi: 10.1097/MD.0000000000022093. PMID: 32925751; PMCID: PMC7489591.
132. Fiorella C, Lorna G. COVID-19 in a multiple sclerosis (MS) patient treated with alemtuzumab: Insight to the immune response after COVID. Mult Scler Relat Disord. 2020 Nov;46:102447. doi: 10.1016/j.msard.2020.102447. Epub 2020 Aug 10. PMID: 32835901; PMCID: PMC7416707.

133. Memish ZA, Faqihi F, Alharthy A, Alqahtani SA, Karakitsos D. Plasma exchange in the treatment of complex COVID-19-related critical illness: controversies and perspectives. Int J Antimicrob Agents. 2021 Feb;57(2):106273. doi: 10.1016/j.ijantimicag.2020.106273. Epub 2020 Dec 25. PMID: 33370568; PMCID: PMC7834644.
134. Abu-Raddad LJ, Chemaitelly H, Malek JA, Ahmed AA, Mohamoud YA, Younuskunju S, Ayoub HH, Al Kanaani Z, Al Khal A, Al Kuwari E, Butt AA, Coyle P, Jeremijenko A, Kaleeckal AH, Latif AN, Shaik RM, Rahim HFA, Yassine HM, Al Kuwari MG, Al Romaihi HE, Al-Thani MH, Bertollini R. Assessment of the risk of SARS-CoV-2 reinfection in an intense re-exposure setting. Clin Infect Dis. 2020 Dec 14:ciaa1846. doi: 10.1093/cid/ciaa1846. Epub ahead of print. PMID: 33315061; PMCID: PMC7799253.
135. Ogega CO, Skinner NE, Blair PW, Park HS, Littlefield K, Ganesan A, Ladiwala P, Antar AA, Ray SC, Betenbaugh MJ, Pekosz A, Klein S, Manabe YC, Cox AL, Bailey JR. Durable SARS-CoV-2 B cell immunity after mild or severe disease. medRxiv [Preprint]. 2020 Oct 30:2020.10.28.20220996. doi: 10.1101/2020.10.28.20220996. PMID: 33140070; PMCID: PMC7605583.
136. Gulati K, Prendecki M, Clarke C, Willicombe M, McAdoo S. COVID-19 Reinfection in a Patient Receiving Immunosuppression for ANCA-associated Vasculitis. Arthritis Rheumatol. 2021 Jan 29. doi: 10.1002/art.41671. Epub ahead of print. PMID: 33512093.
137. Verhagen MD, Brazel DM, Dowd JB, Kashnitsky I, Mills MC. Forecasting spatial, socioeconomic and demographic variation in COVID-19 health care demand in England and Wales. BMC Med. 2020 Jun 29;18(1):203. doi: 10.1186/s12916-020-01646-2. PMID: 32594909; PMCID: PMC7321716.
138. Abdallah H, Porterfield F, Fajgenbaum D. Symptomatic relapse and long-term sequelae of COVID-19 in a previously healthy 30-year-old man. BMJ Case Rep. 2020 Dec 13;13(12):e239825. doi: 10.1136/bcr-2020-239825. PMID: 33318288; PMCID: PMC7736956.
139. McDade TW, McNally EM, Zelikovich AS, D'Aquila R, Mustanski B, Miller A, Vaught LA, Reiser NL, Bogdanovic E, Fallon KS, Demonbreun AR. High seroprevalence for SARS-CoV-2 among household members of essential workers detected using a dried blood spot assay. PLoS One. 2020 Aug 14;15(8):e0237833. doi: 10.1371/journal.pone.0237833. PMID: 32797108; PMCID: PMC7428174.
140. Lumley SF, O'Donnell D, Stoesser NE, Matthews PC, Howarth A, Hatch SB, Marsden BD, Cox S, James T, Warren F, Peck LJ, Ritter TG, de Toledo Z, Warren L, Axten D, Cornall RJ, Jones EY, Stuart DI, Screaton G, Ebner D, Hoosdally S, Chand M, Crook DW, O'Donnell AM, Conlon CP, Pouwels KB, Walker AS, Peto TEA, Hopkins S, Walker TM, Jeffery K, Eyre DW; Oxford University Hospitals Staff Testing Group. Antibody Status and Incidence of SARS-CoV-2 Infection in Health Care Workers. N Engl J Med. 2020 Dec 23:NEJMoa2034545. doi: 10.1056/NEJMoa2034545. Epub ahead of print. PMID: 33369366; PMCID: PMC7781098.
141. Kassa SM, Njagarah JBH, Terefe YA. Analysis of the mitigation strategies for COVID-19: From mathematical modelling perspective. Chaos Solitons Fractals. 2020 Sep;138:109968. doi: 10.1016/j.chaos.2020.109968. Epub 2020 Jun 5. PMID: 32536760; PMCID: PMC7274644.
142. Rihan FA, Alsakaji HJ, Rajivganthi C. Stochastic SIRC epidemic model with time-delay for COVID-19. Adv Differ Equ. 2020;2020(1):502. doi: 10.1186/s13662-020-02964-8. Epub 2020 Sep 18. PMID: 32963509; PMCID: PMC7499021.
143. Simmonds P, Williams S, Harvala H. Understanding the outcomes of COVID-19 - does the current model of an acute respiratory infection really fit? J Gen Virol. 2020 Dec 17. doi: 10.1099/jgv.0.001545. Epub ahead of print. PMID: 33331810.
144. Li J, Zhang C, Wu Z, Wang G, Zhao H. The Mechanism and Clinical Outcome of patients with Corona Virus Disease 2019 Whose Nucleic Acid Test has changed from negative to positive, and the therapeutic efficacy of Favipiravir: A structured summary of a study protocol for a randomised controlled trial. Trials. 2020 Jun 5;21(1):488. doi: 10.1186/s13063-020-04430-y. PMID: 32503657; PMCID: PMC7273382.
145. Selhorst P, Van Ierssel S, Michiels J, Mariën J, Bartholomeeusen K, Dirinck E, Vandamme S, Jansens H, Ariën KK. Symptomatic SARS-CoV-2 reinfection of a health care worker in a Belgian nosocomial outbreak despite primary neutralizing antibody response. Clin Infect Dis. 2020 Dec 14:ciaa1850. doi: 10.1093/cid/ciaa1850. Epub ahead of print. PMID: 33315049; PMCID: PMC7799230.
146. Colson P, Finaud M, Levy N, Lagier JC, Raoult D. Evidence of SARS-CoV-2 re-infection with a different genotype. J Infect. 2020 Nov 15:S0163-4453(20)30706-4. doi: 10.1016/j.jinf.2020.11.011. Epub ahead of print. PMID: 33207255; PMCID: PMC7666873.

147. Dobi A, Sandenon Seteyen AL, Lalarizo Rakoto M, Lebeau G, Vagner D, Frumence É, Giry C, Septembre-Malaterre A, Raffray L, Gasque P. Serological Surveillance of COVID-19 Hospitalized Patients in Réunion Island (France) Revealed that Specific Immunoglobulin G Are Rapidly Vanishing in Severe Cases. J Clin Med. 2020 Nov 27;9(12):3847. doi: 10.3390/jcm9123847. PMID: 33260801; PMCID: PMC7761058.
148. Legros V, Denolly S, Vogrig M, Boson B, Siret E, Rigaill J, Pillet S, Grattard F, Gonzalo S, Verhoeven P, Allatif O, Berthelot P, Pélissier C, Thiery G, Botelho-Nevers E, Millet G, Morel J, Paul S, Walzer T, Cosset FL, Bourlet T, Pozzetto B. A longitudinal study of SARS-CoV-2-infected patients reveals a high correlation between neutralizing antibodies and COVID-19 severity. Cell Mol Immunol. 2021 Feb;18(2):318-327. doi: 10.1038/s41423-020-00588-2. Epub 2021 Jan 6. PMID: 33408342; PMCID: PMC7786875.
149. AlFehaidi A, Ahmad SA, Hamed E. SARS-CoV-2 re-infection: a case report from Qatar. J Infect. 2020 Oct 25:S0163-4453(20)30677-0. doi: 10.1016/j.jinf.2020.10.019. Epub ahead of print. PMID: 33115660; PMCID: PMC7585726.
150. Boonyaratanakornkit J, Morishima C, Selke S, Zamora D, McGuffin S, Shapiro AE, Campbell VL, McClurkan CL, Jing L, Gross R, Liang J, Postnikova E, Mazur S, Chaudhary A, Das MK, Fink SL, Bryan A, Greninger AL, Jerome KR, Holbrook MR, Gernsheimer TB, Wener MH, Wald A, Koelle DM. Clinical, laboratory, and temporal predictors of neutralizing antibodies to SARS-CoV-2 after COVID-19. medRxiv [Preprint]. 2020 Oct 8:2020.10.06.20207472. doi: 10.1101/2020.10.06.20207472. Update in: J Clin Invest. 2020 Dec 15;: PMID: 33052361; PMCID: PMC7553186.
151. Harrington D, Kele B, Pereira S, Couto-Parada X, Riddell A, Forbes S, Dobbie H, Cutino-Moguel T. Confirmed Reinfection with SARS-CoV-2 Variant VOC-202012/01. Clin Infect Dis. 2021 Jan 9:ciab014. doi: 10.1093/cid/ciab014. Epub ahead of print. PMID: 33421056.
152. Mulder M, van der Vegt DSJM, Oude Munnink BB, GeurtsvanKessel CH, van de Bovenkamp J, Sikkema RS, Jacobs EMG, Koopmans MPG, Wegdam-Blans MCA. Reinfection of SARS-CoV-2 in an immunocompromised patient: a case report. Clin Infect Dis. 2020 Oct 9:ciaa1538. doi: 10.1093/cid/ciaa1538. Epub ahead of print. PMID: 33043962; PMCID: PMC7665355.
153. Kim AY, Gandhi RT. Re-infection with SARS-CoV-2: What Goes Around May Come Back Around. Clin Infect Dis. 2020 Oct 9:ciaa1541. doi: 10.1093/cid/ciaa1541. Epub ahead of print. PMID: 33035308; PMCID: PMC7665341.
154. Roy S. COVID-19 Reinfection: Myth or Truth? SN Compr Clin Med. 2020 May 29:1-4. doi: 10.1007/s42399-020-00335-8. Epub ahead of print. PMID: 32838134; PMCID: PMC7255905.
155. Gousseff M, Penot P, Gallay L, Batisse D, Benech N, Bouiller K, Collarino R, Conrad A, Slama D, Joseph C, Lemaignen A, Lescure FX, Levy B, Mahevas M, Pozzetto B, Vignier N, Wyplosz B, Salmon D, Goehringer F, Botelho-Nevers E; in behalf of the COCOREC study group. Clinical recurrences of COVID-19 symptoms after recovery: Viral relapse, reinfection or inflammatory rebound? J Infect. 2020 Nov;81(5):816-846. doi: 10.1016/j.jinf.2020.06.073. Epub 2020 Jun 30. PMID: 32619697; PMCID: PMC7326402.
156. Ota M. Will we see protection or reinfection in COVID-19? Nat Rev Immunol. 2020 Jun;20(6):351. doi: 10.1038/s41577-020-0316-3. PMID: 32303697; PMCID: PMC7186928.
157. Madan M, Kunal S. COVID-19 reinfection or relapse: an intriguing dilemma. Clin Rheumatol. 2020 Nov;39(11):3189. doi: 10.1007/s10067-020-05427-3. Epub 2020 Sep 26. PMID: 32980985; PMCID: PMC7519850.
158. Alizargar J. Risk of reactivation or reinfection of novel coronavirus (COVID-19). J Formos Med Assoc. 2020 Jun;119(6):1123. doi: 10.1016/j.jfma.2020.04.013. Epub 2020 Apr 23. PMID: 32340768; PMCID: PMC7177092.
159. Law SK, Leung AWN, Xu C. Is reinfection possible after recovery from COVID-19? Hong Kong Med J. 2020 Jun;26(3):264-265. doi: 10.12809/hkmj208601. Epub 2020 Jun 5. PMID: 32536614.
160. Selvaraj V, Herman K, Dapaah-Afriyie K. Severe, Symptomatic Reinfection in a Patient with COVID-19. R I Med J (2013). 2020 Nov 9;103(10):24-26. PMID: 33172223.
161. Jabbari P, Rezaei N. With Risk of Reinfection, Is COVID-19 Here to Stay? Disaster Med Public Health Prep. 2020 Aug;14(4):e33. doi: 10.1017/dmp.2020.274. Epub 2020 Jul 27. PMID: 32713383; PMCID: PMC7445454.
162. Victor Okhuese A. Estimation of the Probability of Reinfection With COVID-19 by the Susceptible-Exposed-Infectious-Removed-Undetectable-Susceptible Model. JMIR Public Health Surveill. 2020 May 13;6(2):e19097. doi: 10.2196/19097. PMID: 32369029; PMCID: PMC7223428.

163. Falahi S, Kenarkoohi A. COVID-19 reinfection: prolonged shedding or true reinfection? New Microbes New Infect. 2020 Nov;38:100812. doi: 10.1016/j.nmni.2020.100812. Epub 2020 Nov 12. PMID: 33200033; PMCID: PMC7657875.
164. Kang H, Wang Y, Tong Z, Liu X. Retest positive for SARS-CoV-2 RNA of "recovered" patients with COVID-19: Persistence, sampling issues, or re-infection? J Med Virol. 2020 Nov;92(11):2263-2265. doi: 10.1002/jmv.26114. Epub 2020 Jun 9. PMID: 32492212; PMCID: PMC7300489.
165. West J, Everden S, Nikitas N. A case of COVID-19 reinfection in the UK. Clin Med (Lond). 2021 Jan;21(1):e52-e53. doi: 10.7861/clinmed.2020-0912. Epub 2020 Dec 10. PMID: 33303623; PMCID: PMC7850175.
166. To KK, Hung IF, Chan KH, Yuan S, To WK, Tsang DN, Cheng VC, Chen Z, Kok KH, Yuen KY. Serum antibody profile of a patient with COVID-19 reinfection. Clin Infect Dis. 2020 Sep 23:ciaa1368. doi: 10.1093/cid/ciaa1368. Epub ahead of print. PMID: 32966566; PMCID: PMC7543314.
167. Bonifácio LP, Pereira APS, Araújo DCAE, Balbão VDMP, Fonseca BALD, Passos ADC, Bellissimo-Rodrigues F. Are SARS-CoV-2 reinfection and Covid-19 recurrence possible? a case report from Brazil. Rev Soc Bras Med Trop. 2020 Sep 18;53:e20200619. doi: 10.1590/0037-8682-0619-2020. PMID: 32965458; PMCID: PMC7508196.
168. COVID-19 reinfection: are we ready for winter? EBioMedicine. 2020 Dec;62:103173. doi: 10.1016/j.ebiom.2020.103173. PMID: 33308709; PMCID: PMC7728820.
169. Hoang VT, Dao TL, Gautret P. Recurrence of positive SARS-CoV-2 in patients recovered from COVID-19. J Med Virol. 2020 Nov;92(11):2366-2367. doi: 10.1002/jmv.26056. Epub 2020 Jul 11. PMID: 32449789; PMCID: PMC7280660.
170. Torres DA, Ribeiro LDCB, Riello APFL, Horovitz DDG, Pinto LFR, Croda J. Reinfection of COVID-19 after 3 months with a distinct and more aggressive clinical presentation: Case report. J Med Virol. 2020 Oct 28. doi: 10.1002/jmv.26637. Epub ahead of print. PMID: 33112002.
171. Chen Z, John Wherry E. T cell responses in patients with COVID-19. Nat Rev Immunol. 2020 Sep;20(9):529-536. doi: 10.1038/s41577-020-0402-6. Epub 2020 Jul 29. PMID: 32728222; PMCID: PMC7389156.
172. Chung YH, Beiss V, Fiering SN, Steinmetz NF. COVID-19 Vaccine Frontrunners and Their Nanotechnology Design. ACS Nano. 2020 Oct 27;14(10):12522-12537. doi: 10.1021/acsnano.0c07197. Epub 2020 Oct 9. PMID: 33034449; PMCID: PMC7553041.
173. SeyedAlinaghi S, Oliaei S, Kianzad S, Afsahi AM, MohsseniPour M, Barzegary A, Mirzapour P, Behnezhad F, Noori T, Mehraeen E, Dadras O, Voltarelli F, Sabatier JM. Reinfection risk of novel coronavirus (COVID-19): A systematic review of current evidence. World J Virol. 2020 Dec 15;9(5):79-90. doi: 10.5501/wjv.v9.i5.79. PMID: 33363000; PMCID: PMC7747024.
174. York A. Can COVID-19 strike twice? Nat Rev Microbiol. 2020 Sep;18(9):477. doi: 10.1038/s41579-020-0424-x. PMID: 32690876; PMCID: PMC7369566.
175. Parry J. Covid-19: Hong Kong scientists report first confirmed case of reinfection. BMJ. 2020 Aug 26;370:m3340. doi: 10.1136/bmj.m3340. PMID: 32847834.
176. Dao TL, Hoang VT, Gautret P. Recurrence of SARS-CoV-2 viral RNA in recovered COVID-19 patients: a narrative review. Eur J Clin Microbiol Infect Dis. 2021 Jan;40(1):13-25. doi: 10.1007/s10096-020-04088-z. Epub 2020 Oct 28. PMID: 33113040; PMCID: PMC7592450.
177. Elrashdy F, Aljaddawi AA, Redwan EM, Uversky VN. On the potential role of exosomes in the COVID-19 reinfection/reactivation opportunity. J Biomol Struct Dyn. 2020 Jul 9:1-12. doi: 10.1080/07391102.2020.1790426. Epub ahead of print. PMID: 32643586; PMCID: PMC7441802.
178. Zhou L, Liu K, Liu HG. [Cause analysis and treatment strategies of "recurrence" with novel coronavirus pneumonia (COVID-19) patients after discharge from hospital]. Zhonghua Jie He He Hu Xi Za Zhi. 2020 Apr 12;43(4):281-284. Chinese. doi: 10.3760/cma.j.cn112147-20200229-00219. PMID: 32118391.
179. To KK, Hung IF, Ip JD, Chu AW, Chan WM, Tam AR, Fong CH, Yuan S, Tsoi HW, Ng AC, Lee LL, Wan P, Tso E, To WK, Tsang D, Chan KH, Huang JD, Kok KH, Cheng VC, Yuen KY. COVID-19 re-infection by a phylogenetically distinct SARS-coronavirus-2 strain confirmed by whole genome sequencing. Clin Infect Dis. 2020 Aug 25:ciaa1275. doi: 10.1093/cid/ciaa1275. Epub ahead of print. PMID: 32840608; PMCID: PMC7499500.
180. Iwasaki A. What reinfections mean for COVID-19. Lancet Infect Dis. 2021 Jan;21(1):3-5. doi: 10.1016/S1473-3099(20)30783-0. Epub 2020 Oct 12. Erratum in: Lancet Infect Dis. 2020 Nov 6;: PMID: 33058796; PMCID: PMC7550040.

181. Lafaie L, Célarier T, Goethals L, Pozzetto B, Grange S, Ojardias E, Annweiler C, Botelho-Nevers E. Recurrence or Relapse of COVID-19 in Older Patients: A Description of Three Cases. J Am Geriatr Soc. 2020 Oct;68(10):2179-2183. doi: 10.1111/jgs.16728. Epub 2020 Aug 13. PMID: 32638347; PMCID: PMC7361461.
182. Ioannidis JPA. Global perspective of COVID-19 epidemiology for a full-cycle pandemic. Eur J Clin Invest. 2020 Dec;50(12):e13423. doi: 10.1111/eci.13423. Epub 2020 Oct 25. PMID: 33026101; PMCID: PMC7646031.
183. Velikova TV, Kotsev SV, Georgiev DS, Batselova HM. Immunological aspects of COVID-19: What do we know? World J Biol Chem. 2020 Sep 27;11(2):14-29. doi: 10.4331/wjbc.v11.i2.14. PMID: 33024515; PMCID: PMC7520644.
184. Iyengar KP, Jain VK, Ish P. COVID-19 reinfection - An enigmatic public health threat. Monaldi Arch Chest Dis. 2020 Nov 30;90(4). doi: 10.4081/monaldi.2020.1596. PMID: 33305559.
185. Poonia B, Kottilil S. Immune Correlates of COVID-19 Control. Front Immunol. 2020 Sep 29;11:569611. doi: 10.3389/fimmu.2020.569611. PMID: 33133083; PMCID: PMC7550526.
186. Bongiovanni M, Basile F. Re-infection by COVID-19: a real threat for the future management of pandemia? Infect Dis (Lond). 2020 Aug;52(8):581-582. doi: 10.1080/23744235.2020.1769177. Epub 2020 May 21. PMID: 32434442.
187. Lu J, Peng J, Xiong Q, Liu Z, Lin H, Tan X, Kang M, Yuan R, Zeng L, Zhou P, Liang C, Yi L, du Plessis L, Song T, Ma W, Sun J, Pybus OG, Ke C. Clinical, immunological and virological characterization of COVID-19 patients that test re-positive for SARS-CoV-2 by RT-PCR. EBioMedicine. 2020 Sep;59:102960. doi: 10.1016/j.ebiom.2020.102960. Epub 2020 Aug 24. PMID: 32853988; PMCID: PMC7444471.
188. Biswas A, Bhattacharjee U, Chakrabarti AK, Tewari DN, Banu H, Dutta S. Emergence of Novel Coronavirus and COVID-19: whether to stay or die out? Crit Rev Microbiol. 2020 Mar;46(2):182-193. doi: 10.1080/1040841X.2020.1739001. Epub 2020 Apr 13. PMID: 32282268; PMCID: PMC7157960.
189. Yamamoto V, Bolanos JF, Fiallos J, Strand SE, Morris K, Shahrokhinia S, Cushing TR, Hopp L, Tiwari A, Hariri R, Sokolov R, Wheeler C, Kaushik A, Elsayegh A, Eliashiv D, Hedrick R, Jafari B, Johnson JP, Khorsandi M, Gonzalez N, Balakhani G, Lahiri S, Ghavidel K, Amaya M, Kloor H, Hussain N, Huang E, Cormier J, Wesson Ashford J, Wang JC, Yaghobian S, Khorrami P, Shamloo B, Moon C, Shadi P, Kateb B. COVID-19: Review of a 21st Century Pandemic from Etiology to Neuro-psychiatric Implications. J Alzheimers Dis. 2020;77(2):459-504. doi: 10.3233/JAD-200831. PMID: 32925078; PMCID: PMC7592693.
190. Deng W, Bao L, Liu J, Xiao C, Liu J, Xue J, Lv Q, Qi F, Gao H, Yu P, Xu Y, Qu Y, Li F, Xiang Z, Yu H, Gong S, Liu M, Wang G, Wang S, Song Z, Liu Y, Zhao W, Han Y, Zhao L, Liu X, Wei Q, Qin C. Primary exposure to SARS-CoV-2 protects against reinfection in rhesus macaques. Science. 2020 Aug 14;369(6505):818-823. doi: 10.1126/science.abc5343. Epub 2020 Jul 2. PMID: 32616673; PMCID: PMC7402625.
191. Gray CM, Peter J, Mendelson M, Madhi S, Blackburn JM. COVID-19 antibody testing: From hype to immunological reality. S Afr Med J. 2020 Jul 27;110(9):837-841. PMID: 32880263.
192. Garcia-Beltran WF, Lam EC, Astudillo MG, Yang D, Miller TE, Feldman J, Hauser BM, Caradonna TM, Clayton KL, Nitido AD, Murali MR, Alter G, Charles RC, Dighe A, Branda JA, Lennerz JK, Lingwood D, Schmidt AG, Iafrate AJ, Balazs AB. COVID-19-neutralizing antibodies predict disease severity and survival. Cell. 2021 Jan 21;184(2):476-488.e11. doi: 10.1016/j.cell.2020.12.015. Epub 2020 Dec 15. PMID: 33412089; PMCID: PMC7837114.
193. Kellam P, Barclay W. The dynamics of humoral immune responses following SARS-CoV-2 infection and the potential for reinfection. J Gen Virol. 2020 Aug;101(8):791-797. doi: 10.1099/jgv.0.001439. PMID: 32430094; PMCID: PMC7641391.
194. Lerner AM, Eisinger RW, Lowy DR, Petersen LR, Humes R, Hepburn M, Cassetti MC. The COVID-19 Serology Studies Workshop: Recommendations and Challenges. Immunity. 2020 Jul 14;53(1):1-5. doi: 10.1016/j.immuni.2020.06.012. Epub 2020 Jun 23. PMID: 32610080; PMCID: PMC7309808.
195. Jain A, Kaur J, Rai AK, Pandey AK. Anosmia: A Clinical Indicator of COVID-19 Reinfection. Ear Nose Throat J. 2020 Dec 9:145561320978169. doi: 10.1177/0145561320978169. Epub ahead of print. PMID: 33295221.

196. Baker D, Amor S, Kang AS, Schmierer K, Giovannoni G. The underpinning biology relating to multiple sclerosis disease modifying treatments during the COVID-19 pandemic. Mult Scler Relat Disord. 2020 Aug;43:102174. doi: 10.1016/j.msard.2020.102174. Epub 2020 May 12. PMID: 32464584; PMCID: PMC7214323.
197. Arteaga-Livias K, Panduro-Correa V, Pinzas-Acosta K, Perez-Abad L, Pecho-Silva S, Espinoza-Sánchez F, Dámaso-Mata B, Rodriguez-Morales AJ. COVID-19 reinfection? A suspected case in a Peruvian patient. Travel Med Infect Dis. 2020 Dec 8;39:101947. doi: 10.1016/j.tmaid.2020.101947. Epub ahead of print. PMID: 33307196; PMCID: PMC7723440.
198. Bongiovanni M. COVID-19 re-infection in an healthcare worker. J Med Virol. 2020 Sep 29:10.1002/jmv.26565. doi: 10.1002/jmv.26565. Epub ahead of print. PMID: 32990954; PMCID: PMC7537129.
199. Teslya A, Pham TM, Godijk NG, Kretzschmar ME, Bootsma MCJ, Rozhnova G. Impact of self-imposed prevention measures and short-term government-imposed social distancing on mitigating and delaying a COVID-19 epidemic: A modelling study. PLoS Med. 2020 Jul 21;17(7):e1003166. doi: 10.1371/journal.pmed.1003166. Erratum in: PLoS Med. 2020 Dec 4;17(12):e1003499. PMID: 32692736; PMCID: PMC7373263.
200. Krishna E, Pathak VK, Prasad R, Jose H, Kumar MM. COVID-19 reinfection: Linked Possibilities and future outlook. J Family Med Prim Care. 2020 Nov 30;9(11):5445-5449. doi: 10.4103/jfmpc.jfmpc_1672_20. PMID: 33532377; PMCID: PMC7842419.
201. Duggan NM, Ludy SM, Shannon BC, Reisner AT, Wilcox SR. Is novel coronavirus 2019 reinfection possible? Interpreting dynamic SARS-CoV-2 test results. Am J Emerg Med. 2021 Jan;39:256.e1-256.e3. doi: 10.1016/j.ajem.2020.06.079. Epub 2020 Jul 4. PMID: 32703607; PMCID: PMC7335242.
202. Zhang B, Liu S, Dong Y, Zhang L, Zhong Q, Zou Y, Zhang S. Positive rectal swabs in young patients recovered from coronavirus disease 2019 (COVID-19). J Infect. 2020 Aug;81(2):e49-e52. doi: 10.1016/j.jinf.2020.04.023. Epub 2020 Apr 23. PMID: 32335176; PMCID: PMC7177113.
203. Nachmias V, Fusman R, Mann S, Koren G. The first case of documented Covid-19 reinfection in Israel. IDCases. 2020;22:e00970. doi: 10.1016/j.idcr.2020.e00970. Epub 2020 Oct 1. PMID: 33029476; PMCID: PMC7528892.
204. Arafkas M, Khosrawipour T, Kocbach P, Zielinski K, Schubert J, Mikolajczyk A, Celinska M, Khosrawipour V. Current meta-analysis does not support the possibility of COVID-19 reinfections. J Med Virol. 2020 Sep 8. doi: 10.1002/jmv.26496. Epub ahead of print. PMID: 32897549.
205. Nainu F, Abidin RS, Bahar MA, Frediansyah A, Emran TB, Rabaan AA, Dhama K, Harapan H. SARS-CoV-2 reinfection and implications for vaccine development. Hum Vaccin Immunother. 2020 Dec 1;16(12):3061-3073. doi: 10.1080/21645515.2020.1830683. PMID: 33393854.
206. Edridge AWD, Kaczorowska J, Hoste ACR, Bakker M, Klein M, Loens K, Jebbink MF, Matser A, Kinsella CM, Rueda P, Ieven M, Goossens H, Prins M, Sastre P, Deijs M, van der Hoek L. Seasonal coronavirus protective immunity is short-lasting. Nat Med. 2020 Nov;26(11):1691-1693. doi: 10.1038/s41591-020-1083-1. Epub 2020 Sep 14. PMID: 32929268.
207. Bongiovanni M, Vignati M, Giuliani G, Manes G, Arienti S, Pelucchi L, Cattaneo N, Bodini BD, Clerici D, Rosa F, Pellegrini L, Schettino M, Picascia D, Bini F. The dilemma of COVID-19 recurrence after clinical recovery. J Infect. 2020 Dec;81(6):979-997. doi: 10.1016/j.jinf.2020.08.019. Epub 2020 Aug 15. PMID: 32810521; PMCID: PMC7428731.
208. Zhou W, Xu X, Chang Z, Wang H, Zhong X, Tong X, Liu T, Li Y. The dynamic changes of serum IgM and IgG against SARS-CoV-2 in patients with COVID-19. J Med Virol. 2021 Feb;93(2):924-933. doi: 10.1002/jmv.26353. Epub 2020 Sep 28. PMID: 32706425; PMCID: PMC7404900.
209. Malkov E. Simulation of coronavirus disease 2019 (COVID-19) scenarios with possibility of reinfection. Chaos Solitons Fractals. 2020 Oct;139:110296. doi: 10.1016/j.chaos.2020.110296. Epub 2020 Sep 18. PMID: 32982082; PMCID: PMC7500883.
210. Ching L, Chang SP, Nerurkar VR. COVID-19 Special Column: Principles Behind the Technology for Detecting SARS-CoV-2, the Cause of COVID-19. Hawaii J Health Soc Welf. 2020 May 1;79(5):136-142. PMID: 32432217; PMCID: PMC7226308.
211. Harari S, Vitacca M. COVID-19 spread: The Italian case. Respir Med Res. 2020 Nov;78:100771. doi: 10.1016/j.resmer.2020.100771. Epub 2020 May 23. PMID: 32502937; PMCID: PMC7244411.
212. Wiersinga WJ, de Bree GJ. Re-infectie met SARS-CoV-2 [Covid-19 reinfections: what is the clinical relevance?]. Ned Tijdschr Geneeskd. 2020 Dec 3;164:D5640. Dutch. PMID: 33332056.

213. Hanif M, Haider MA, Ali MJ, Naz S, Sundas F. Reinfection of COVID-19 in Pakistan: A First Case Report. Cureus. 2020 Oct 26;12(10):e11176. doi: 10.7759/cureus.11176. PMID: 33262913; PMCID: PMC7689968.
214. Bentivegna E, Sentimentale A, Luciani M, Speranza ML, Guerritore L, Martelletti P. New IgM seroconversion and positive RT-PCR test after exposure to the virus in recovered COVID-19 patient. J Med Virol. 2020 Jun 11:10.1002/jmv.26160. doi: 10.1002/jmv.26160. Epub ahead of print. PMID: 32525558; PMCID: PMC7300757.
215. Mumoli N, Vitale J, Mazzone A. Clinical immunity in discharged medical patients with COVID-19. Int J Infect Dis. 2020 Oct;99:229-230. doi: 10.1016/j.ijid.2020.07.065. Epub 2020 Aug 7. PMID: 32771639; PMCID: PMC7411424.
216. Lechien JR, Chiesa-Estomba CM, Vaira LA, Saussez S, Hans S. COVID-19 Reinfection and Second Episodes of Olfactory and Gustatory Dysfunctions: Report of First Cases. Ear Nose Throat J. 2020 Nov 10:145561320970105. doi: 10.1177/0145561320970105. Epub ahead of print. PMID: 33170036.
217. Abid MA, Nunley L, Abid MB. Could Coronavirus Disease 2019 (COVID-19) Render Natural Immunity to Re-infections? A Spotlight on the Therapeutic Pipeline. Front Immunol. 2020 Jun 5;11:1294. doi: 10.3389/fimmu.2020.01294. PMID: 32582221; PMCID: PMC7290157.
218. Stokel-Walker C. What we know about covid-19 reinfection so far. BMJ. 2021 Jan 19;372:n99. doi: 10.1136/bmj.n99. PMID: 33468457.
219. Sen MK, Gupta N, Yadav SR, Kumar R, Singh B, Ish P. Contentious Issue in Recurrent COVID-19 Infection: Reactivation or Reinfection. Turk Thorac J. 2020 Nov;21(6):463-466. doi: 10.5152/TurkThoracJ.2020.20164. Epub 2020 Nov 1. PMID: 33352106; PMCID: PMC7752104.
220. Griffin S. Covid-19: Antibodies protect against reinfection for at least six months, study finds. BMJ. 2020 Dec 30;371:m4961. doi: 10.1136/bmj.m4961. PMID: 33380408.
221. Coish JM, MacNeil AJ. Out of the frying pan and into the fire? Due diligence warranted for ADE in COVID-19. Microbes Infect. 2020 Oct;22(9):405-406. doi: 10.1016/j.micinf.2020.06.006. Epub 2020 Jun 24. PMID: 32590062; PMCID: PMC7311339.
222. Batra R, Olivieri LG, Rubin D, Vallari A, Pearce S, Olivo A, Prostko J, Nebbia G, Douthwaite S, Rodgers M, Cloherty G. A comparative evaluation between the Abbott Panbio™ COVID-19 IgG/IgM rapid test device and Abbott Architect™ SARS CoV-2 IgG assay. J Clin Virol. 2020 Nov;132:104645. doi: 10.1016/j.jcv.2020.104645. Epub 2020 Sep 16. PMID: 32961429; PMCID: PMC7493757.
223. Zhou B, Kojima S, Kawamoto A, Fukushima M. COVID-19 pathogenesis, prognostic factors, and treatment strategy: Urgent recommendations. J Med Virol. 2020 Dec 23. doi: 10.1002/jmv.26754. Epub ahead of print. PMID: 33368358.
224. Martín Enguix D, Aguirre Rodríguez JC, Sánchez Cambronero M, Hidalgo Rodríguez A. PCR para COVID-19 positiva, luego negativa y otra vez positiva ¿Reinfección a los 55 días? [PCR for COVID-19 positive, then negative and again positive Reinfection at 55 days?]. Semergen. 2020 Dec 23:S1138-3593(20)30410-X. Spanish. doi: 10.1016/j.semerg.2020.12.001. Epub ahead of print. PMID: 33478840; PMCID: PMC7755571.
225. Lancman G, Mascarenhas J, Bar-Natan M. Severe COVID-19 virus reactivation following treatment for B cell acute lymphoblastic leukemia. J Hematol Oncol. 2020 Oct 2;13(1):131. doi: 10.1186/s13045-020-00968-1. PMID: 33008453; PMCID: PMC7531062.
226. Song KH, Kim DM, Lee H, Ham SY, Oh SM, Jeong H, Jung J, Kang CK, Park JY, Kang YM, Kim JY, Park JS, Park KU, Kim ES, Kim HB. Dynamics of viral load and anti-SARS-CoV-2 antibodies in patients with positive RT-PCR results after recovery from COVID-19. Korean J Intern Med. 2021 Jan;36(1):11-14. doi: 10.3904/kjim.2020.325. Epub 2020 Nov 25. PMID: 32972123; PMCID: PMC7820639.
227. Sharma R, Sardar S, Mohammad Arshad A, Ata F, Zara S, Munir W. A Patient with Asymptomatic SARS-CoV-2 Infection Who Presented 86 Days Later with COVID-19 Pneumonia Possibly Due to Reinfection with SARS-CoV-2. Am J Case Rep. 2020 Dec 1;21:e927154. doi: 10.12659/AJCR.927154. PMID: 33257644; PMCID: PMC7718490.
228. Zapor M. Persistent Detection and Infectious Potential of SARS-CoV-2 Virus in Clinical Specimens from COVID-19 Patients. Viruses. 2020 Dec 3;12(12):1384. doi: 10.3390/v12121384. PMID: 33287245; PMCID: PMC7761721.
229. Danchin A, Turinici G. Immunity after COVID-19: Protection or sensitization? Math Biosci. 2021 Jan;331:108499. doi: 10.1016/j.mbs.2020.108499. Epub 2020 Oct 28. PMID: 33129826; PMCID: PMC7598904.
